# Electrochemical Properties and Structure of Membranes from Perfluorinated Copolymers Modified with Nanodiamonds

**DOI:** 10.3390/membranes13110850

**Published:** 2023-10-25

**Authors:** Vasily T. Lebedev, Yuri V. Kulvelis, Alexandr V. Shvidchenko, Oleg N. Primachenko, Alexei S. Odinokov, Elena A. Marinenko, Alexander I. Kuklin, Oleksandr I. Ivankov

**Affiliations:** 1 Petersburg Nuclear Physics Institute Named by B.P. Konstantinov of National Research Center “Kurchatov Institute”, 188300 Gatchina, Russia; 2 Ioffe Institute, 194021 St. Petersburg, Russia; avshvid@mail.ioffe.ru; 3 Institute of Macromolecular Compounds, Russian Academy of Sciences, 199004 St. Petersburg, Russia; alex-prima@mail.ru (O.N.P.); emarinenkospb@gmail.com (E.A.M.); 4 Russian Research Center of Applied Chemistry, 193232 St. Petersburg, Russia; oas9@mail.ru; 5 Frank Laboratory of Neutron Physics, Joint Institute for Nuclear Research, 141980 Dubna, Russia; alexander.iw.kuklin@gmail.com (A.I.K.); ivankov@jinr.ru (O.I.I.)

**Keywords:** nanodiamonds, perfluorosulfonic acid membrane, proton exchange membrane, proton conductivity, small-angle neutron scattering, short side chain, ion channel, composite structure

## Abstract

In this study, we aimed to design and research proton-conducting membranes based on Aquivion^®^-type material that had been modified with detonation nanodiamonds (particle size 4–5 nm, 0.25–5.0 wt. %). These nanodiamonds carried different functional groups (H, OH, COOH, F) that provided the hydrophilicity of the diamond surface with positive or negative potential, or that strengthened the hydrophobicity of the diamonds. These variations in diamond properties allowed us to find ways to improve the composite structure so as to achieve better ion conductivity. For this purpose, we prepared three series of membrane films by first casting solutions of perfluorinated Aquivion^®^-type copolymers with short side chains mixed with diamonds dispersed on solid substrates. Then, we removed the solvent and the membranes were structurally stabilized during thermal treatment and transformed into their final form with –SO_3_H ionic groups. We found that the diamonds with a hydrogen-saturated surface, with a positive charge in aqueous media, contributed to the increase in proton conductivity of membranes to a greater rate. Meanwhile, a more developed conducting diamond-copolymer interface was formed due to electrostatic attraction to the sulfonic acid groups of the copolymer than in the case of diamonds grafted with negatively charged carboxyls, similar to sulfonic groups of the copolymer. The modification of membranes with fluorinated diamonds led to a 5-fold decrease in the conductivity of the composite, even when only a fraction of diamonds of 1 wt. % were used, which was explained by the disruption in the connectivity of ion channels during the interaction of such diamonds mainly with fluorocarbon chains of the copolymer. We discussed the specifics of the mechanism of conductivity in composites with various diamonds in connection with structural data obtained in neutron scattering experiments on dry membranes, as well as ideas about the formation of cylindrical micelles with central ion channels and shells composed of hydrophobic copolymer chains. Finally, the characteristics of the network of ion channels in the composites were found depending on the type and amount of introduced diamonds, and correlations between the structure and conductivity of the membranes were established.

## 1. Introduction

Progress in hydrogen energy is associated with the development of fuel cells using solid electrolytes. Membranes for separating hydrogen fuel from an oxidizer play a key role in providing proton transport at a low electron conductivity. Ion-exchange membranes are also often used in other types of energy storage batteries, especially redox flow batteries, as, nowadays, they have become important for solving the crucial issues regarding renewable energy sources [1,2,3]. Such membranes should have a high proton conductivity, as well as stable electrical and mechanical properties, and be able to withstand oxidative stress at elevated temperatures [4]. To a large extent, these qualities have been achieved in industrial ionomers—perfluorinated copolymers Nafion^®^ and Aquivion^®^, with a similar chemical structure, which differ only in the length of side chains for the –SO_3_H end groups (long and short chains, respectively) [5]. Nevertheless, there are key problems for improving the water-retaining properties and conductivity of such materials at a relatively low humidity at high temperatures.

This makes it necessary to search for methods of modifying perfluorosulfonic acid (PFSA) membranes with nanoparticles (SiO_2_, TiO_2_, ZrO_2_, etc.) [6,7]. These modifications significantly affect the crystallinity and structure of Nafion^®^ and give a gain in water uptake while can slightly decrease ion-exchange capacity. To improve a performance of fuel cells at enhanced temperatures (≥100 °C), there were used Nafion^®^-type membranes [8] filled with ZrO_2_-TiO_2_ (ZT) particles. As a result, under operating conditions (120 °C, relative humidity (RH) 50%, pressure 2 atm.), the electrochemical characteristics of the composites at a ratio of Zr:Ti = 1:3 exceeded those of the Nafion-112^®^, due to higher water uptake as a more important factor for membrane performance at high temperatures than ion-exchange capacity.

A markable fact related to Nafion^®^ matrix filling with SiO_2_, TiO_2_, ZrO_2_ is a reduced conductivity of the composite at low temperatures, but an enhanced conductivity at higher temperatures and lower degrees of membrane moisture, which is the most significant for membrane applications [9].

The problem of water retention can be solved by using the embedded particles with a porous structure, e.g., carbon nanotubes. They provide a gain in conductivity [9], although the effect depends on functionalization type (protonation, hydroxylation, sulfonation etc.) [10,11]. However, such particles are usually larger than clusters of polymeric ionic groups, inverted micelles (~4 nm) and ion channels binding them (~1 nm in diameter) according to Gierke model [12]. These particles cannot be incorporated into polymer conductive structures trend to segregate. To create more perfect membrane composites, tiny particles no larger than the scale of the initial polymer structure are desirable for the formation of hybrid conducting structures.

For these purposes, nanodiamonds resistant to aggressive media detonation (DND, particle size 4–5 nm) with various functional groups on the surface (H, OH, COOH, SO_3_H) are the most suitable [11,13,14,15]. Variations in the type and number of grafted groups make it possible to control the sign and value of the diamond surface potential (30–70 mV) in a hydrated state [15].

In a perfluorinated copolymer, hydrophilic diamonds DND Z+ with a positive surface charge should be predominantly adsorbed in the membrane areas saturated with negatively charged SO_3_H groups, where the formation of hybrid conductive channels is possible if the particle size of the diamond is comparable to the diameter of the aqueous cavities in the membranes, according to Gierke’s channel-cluster model [12]. It is also expected that the copolymer chains can create planar ion channels along diamond facets due to electrostatic attraction to DND Z+ particles. This is supported by the fact that a water-saturated Aquivion^®^-type composite (0.5 wt. % DND Z+) showed an increase in conductivity comparative to the pristine material [16]. On the other hand, Aquivion^®^-type composites with DND Z− diamonds with a negative surface potential did not demonstrate a significant effect due to the mutual repulsion of similarly charged ionic groups and diamonds [17]. In general, the interaction of diamonds (DND Z+, DND Z−) with ionic groups of copolymers should be considered in terms of the formation of multiplets created by ionic groups that are surrounded by hydrophobic chain fragments. These chain fragments are capable of adopting a conformation resembling folded lamellae, which can be combined into micellar structures [18,19].

The key fact here is that in membranes, ionic clusters accumulating water are packed into the shells of hydrophobic polymer chains [20]. Therefore, to describe the conductive network of membranes, combinations of spherical and cylindrical inverted micelles are used, which have water cavities and form bundles in the polymer matrix according to the neutron and X-ray scattering data [21,22,23,24].

The results from the neutron scattering experiments on Nafion^®^ membranes [25,26] have shown they contain cylindrical straight or curved ion channels that can absorb water. Schmidt-Rohr et al. [27] developed a model of a network of cylindrical ion channels in polymer shells with a local orientational order (channel bundles) based on X-ray and neutron scattering data. It was shown that polymer chains have helical conformations, which correspond to cylindrical inverse micelles; this is in agreement with the data on the degree of crystallinity of copolymers. As an alternative model of membranes, Kreuer et al. [28,29] proposed the formation of polymer lamellae structures with narrow two-dimensional diffusion channels (~1 nm thick). A generalized analysis of the membrane morphology [30] led to the concept of a percolation network of ion channels in them in combination with a hydrophobic network of fluorocarbon chains in relation to both the isotropic and oriented membranes when describing the correlations between the structure and transport properties. In composite membranes, the ordering of ionic groups can also be realized as cylindrical [27] or flat channels [28,29]; however, lamellae formation on the surface of modifier particles seems to be preferable for diamonds, as they can create a conductive surface with the adjacent free volume at the polymer interface, particularly when they adopt a folded conformation.

To evaluate the effect of diamonds on conductivity, we considered a thin membrane with a unit surface area and a number (N_ch_) of channels (radius r_ch_) perpendicular to the surface. This composition provides a specific conductivity of film κ = κ_ch_N_ch_(πr_ch_^2^) as being proportional to the specific conductivity (κ_ch_) of a channel. This implies that the conductivity κ = (1/2)S_tch_κ_ch_r_ch_ is proportional to the total area (S_tch_) of the inner surface of the channel per unit volume of the membrane. When diamonds are introduced into the polymer, the area increases due to the diamond surface and adjacent polymer border. Effectively, it provides growth in the number of channels and in the conductivity of the membrane.

A correlation between the growth of the inner surface and conductivity was observed in Aquivion^®^-type membranes modified with DND Z− diamonds (4–5 nm in diameter) grafted with carboxyls [31]. Material strengthening took place for the membranes with diamonds (0.25–1.0 wt. %). At the maximum water uptake, their conductivity increased upon heating (20–50 °C) to a greater extent than that in the pure matrix. The composite (1.0 wt. %) had 1.4 × 10^21^ cm^−3^ SO_3_H groups, a diamond numerical concentration of 1.2 × 10^17^ cm^−3^, and a total surface area of 7.6 × 10^4^ cm^2^/cm^3^. For such a number of groups (~0.3 nm in size), the area of ion channels they covered was ~1.2 × 10^6^ cm^2^/cm^3^. As a result of the presence of diamonds (1 wt. %), the area of the inner surface increased by ~6%, which was similar to the material conductivity enhancement of ~ 8% for the pure copolymer, at 50 °C [31]. An increase in conductivity of ~10% was also observed in the Aquivion^®^-type membranes modified by DND-S diamonds (1 wt. %) grafted with SO_3_H groups at 50 °C [11]. In both cases, the acidic groups created a negative charge on the diamond particles in an aqueous environment, and the formation of a diamond-polymer conductive interface was hampered by the repulsion of components.

Thus, it is promising introduce DND Z+ diamonds, which carry positively charged groups, into membranes, because mutually attracted components stimulate the formation of hybrid ionic channels, and binding these channels to the polymer’s conducting network results in the entire membrane structure becoming more stabilized. Consequently, the membrane structure become more regular and prevents hydrogen fuel crossover. This is supported by neutron scattering data regarding Nafion^®^-type copolymer membranes with long side chains [32]. The introduction of hydrogen-saturated DND Z+ diamonds (0.25–3.1 wt. %, ~4–5 nm particle size) into Nafion^®^-type membranes caused the polymer ionic channel’s radius to expand from 0.7 to 0.8 nm and their surface area to increase by ~15%. It is worth noting that such results are related the copolymer with long side chains, but structural changes in the matrix composed of the Aquivion^®^-type copolymer with short side chains can be different.

For the composites based on Aquivion^®^-type copolymers with DND Z+ diamonds, a detailed analysis of the molecular structure and morphology, raning from several to hundreds of nanometers, has not been carried out. Recent neutron scattering experiments on dry composites with DND Z+ diamonds (0.25–5.0 wt. %) demonstrated well-defined ionomer peaks. The position of the ionomer peak changed minimally with different filler concentrations, and the corresponding period in the channel packing was ~3 nm. This shows that hydrophilic diamonds had a weak effect on the assembly of ionic channels [10]. Furthermore, it is also important to elucidate how strongly hydrophobic diamonds are able to modulate the copolymer structure as they mainly interact with fluorocarbon chains, rather than with ionic groups, as observed in the case of membrane modification with DND Z+ and DND Z− particles. The creation and study of composites with fluorinated diamonds (DND-F) is crucial for a general understanding of the nature of the influence of diamonds on copolymer molecular and supramolecular ordering. 

Until now, the structure of Aquivion^®^-based composites and their conductivity, with a focus on the specific type of diamonds modifiers featuring various surface modifications, have not been studied in detail. The aim of this work is to obtain composites based on the Aquivion^®^-type material using three types of diamonds (DND Z+, DND Z−, and DND-F) and to perform a subsequent comparative analysis of their structure and electrical properties under conditions of varying modifier concentrations (0–5 wt. %) and sample temperatures (20–50 °C).

## 2. Experimental

### 2.1. Materials Obtaining and Characterization

Detonation nanodiamonds (DND, size d_p_ ≈ 4–5 nm with spread δdp/dp ≈ 50%) were produced through detonation synthesis (UDD-STP, Special Design and Technological Bureau “Technolog”, St. Petersburg, Russia), followed by chemical purification (etching in acids HF and HCl). Then, they underwent deagglomeration through annealing and ultrasonic dispersion [13,33,34,35].

After the removal of graphene-like fragments from the surface of the diamond particles, purified crystals were tested using X-ray diffraction; this only showed the diamond phase [34,35]. The diamonds were produced using methods [34,35] that formed stable hydrosols (concentrations ~1% wt.), where the DND Z+ and DND Z− particles had an adjustable (positive or negative) surface potential of ~ 30–70 mV, depending on the annealing technology. A typical transmission electron microscopy (TEM) of the diamond aggregates formed by hydrosol drying is shown in Figure S1. The morphology of diamond powders with various functional groups (H, OH, COOH, F) has already been studied in detail [36,37,38,39,40].

**Figure 1:**
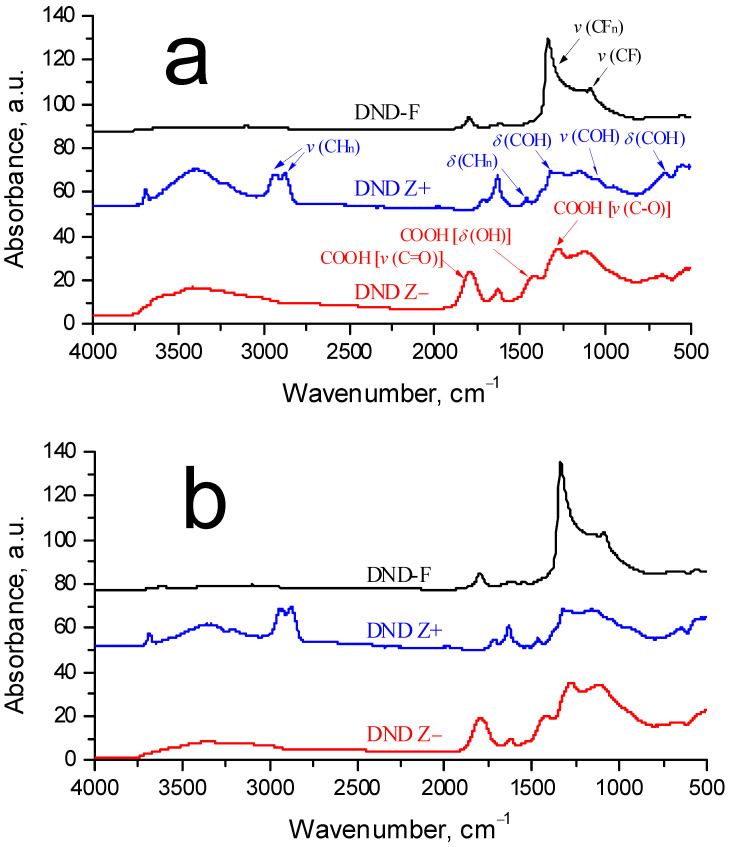
FTIR spectra of diamond nanoparticles of DND Z−, DND Z+, and DND-F before (**a**) and after heating in air at 80 °C for 2 h (**b**).

Diamonds possessing a positive potential (DND Z+) through hydrogenation were produced from purified diamond nanoparticles powders through annealing in a hydrogen flow at 600 °C for 3 h in order to graft H and OH functional groups onto their surfaces. Next, the particles were mixed with deionized water, subjected to intense ultrasonic treatment, and centrifuged (1.8 × 10^4^× g, 40 min) in order to be separated into a fine fraction [34]. Diamonds with a negative potential (DND Z−) were prepared by annealing the purified powder in air (430 °C, 6 h) in order to graft functional COOH groups onto their surfaces [13], using the same subsequent procedures. In addition to DND Z−, DND Z+ diamonds with ionic groups (H, OH, COOH), another type of diamond (DND-F) with grafted fluorine atoms, were prepared [38,39,40]. During the process of modification, molecular fluorine interacted with the surface groups (C–OH, C–H, C=O, COOH) of the diamonds at 450 °C. As a result of group decomposition, C–F bonds were formed when 97% of hydrogen atoms were substituted with fluorine [40]. Stable sols were prepared from fluorinated diamonds in dimethylformamide (DMF) with a solid phase concentration of 0.17 wt. %. In the following tests, DND-F particles were dispersed in ethanol, where their surface potential (negative) was measured. Dynamic light scattering data (Zetasizer Nano ZS analyzer, Malvern Instruments Ltd., Malvern, UK) showed an average particle size distribution of 4–5 nm and the presence of ~20 nm scale aggregates. The FTIR spectra, registered using an infrared Fourier spectrometer InfraLUM FT-08 (Lumex, St. Petersburg, Russia) with a diffuse reflectance infrared Fourier transform (DRIFT) accessory, of the diamond nanoparticles used are presented in Figure 1a. Heating the DND powders in air did not affect the composition of the surface functional groups of particles (Figure 1b). 

Dynamic light scattering data (Zetasizer Nano ZS analyzer, Malvern Instruments Ltd.) showed an average particle size distribution of 4–5 nm and the presence of ~20 nm scale aggregates.

To prepare the composites, a short side chain PFSA Aquivion^®^-type copolymer was synthesized using aqueous emulsion technology [41] through copolymerizing tetrafluoroethylene (TFE) with a perfluorinated sulfonic monomer (perfluoro-3-oxapentenesulfonyl fluoride). The process was conducted in a 0.45 L steel reactor that was maintained at a temperature of 40–60 °C, using an anchor stirrer at a pressure of 0.7–1.3 MPa supplied to the TFE system that was controlled automatically. TFE was supplied directly to the emulsion of the preliminarily prepared sulfonyl fluoride monomer stabilized with a perfluorinated surfactant, namely ammonium perfluorononate.

Here, we used a short side chain copolymer in -SO_2_F form as a precursor, which was obtained through aqueous-emulsion copolymerization of fluoromonomers [41] with an equivalent weight (EW) of 890 g-eq/mol for the SO_2_F groups (melt flow index MFI = 0.80 g/10 min determined on an IIRT-M plastometer at 270 °C, load 2.16 kg, and capillary diameter 2.095 mm).

Hydrolysis of the precursor copolymer was carried out according to the following procedure: Copolymer powder in -SO_2_F form was loaded into a three-neck flask and filled with a solution of 5% LiOH in deionized water (100% excess). The flask was heated to 90 °C while stirring, and the reaction mixture was maintained for 2 h. The resulting copolymer in -SO_3_Li form was filtered and washed three times with deionized water to remove residual LiOH and LiF, until the wash water was neutral. The polymer powder in -SO_3_Li form was dried to a 50% moisture content (by weight). Then, we obtained a dispersion (2 wt. %) of the Aquivion^®^-type copolymer in -SO_3_Li form in dimethylformamide (DMF) using ultrasonic treatment (10 min, emitter power 150 W, frequency 22 kHz).

To modify the Aquivion^®^-type copolymer, a dispersion of DND Z+ in DMF (0.31 wt. %) was used. This was first obtained from the suspension of DND Z+ powder in DMF, followed by centrifugation and separation of the precipitate according to the methods developed at the Ioffe Institute [13,34,35]. When preparing the mixture of polymer with DND Z+, a DMF dispersion of the polymer (2 wt. %) was initially filtered under vacuum (Schott filter, pore size 16–40 μm), followed by the addition of the required amount of 0.31 wt. % of DND Z+ dispersion with mechanical stirring for 30 s, followed by ultrasonic treatment for 10 min.

The formation of Aquivion^®^-type compositional membranes with DND Z+ was carried out using a casting process on a glass substrate in an air chamber at 70–72 °C for 5 h [42]. After obtaining the composite membrane in the -SO_3_Li form of the copolymer, it was converted to a final –SO_3_H form through treatment with 15 wt. % HNO_3_ solution at room temperature and stirring for 2 h. The membrane was then washed in distilled water for 2 h and then in fresh distilled water for 16 h. We performed the conversion of –SO_2_F groups in –SO_3_H using the adapted two-stage procedure [43,44], where the polymer was first subjected to alkaline hydrolysis, and was then acidified using nitric or any other strong acid solution at room temperature. The obtained pristine Aquivion^®^-type membrane and compositional membranes with DND Z+ demonstrated excellent mechanical properties and thermal stability [10].

Similar procedures were used to obtain the composite membranes for the DND Z− and DND-F diamonds, which were functionalized by carboxyl groups or fluorine atoms, respectively. A pure membrane without diamonds was also casted and processed using a similar technique, using only a copolymer solution without the addition of DND. This process yielded a copolymer chain with an equivalent weight (EW) of 890 g-eq/mol per ionic group (–SO_3_H), as indicated by the ion-exchange capacity data. This is optimal for proton-conducting membranes in terms of the balance between electrochemical and physical and mechanical properties. The membrane film of this copolymer had a sufficiently high proton conductivity of 0.145 S/cm at 20 °C and a maximum equilibrium water content of 37.0 wt. %. The same copolymer with EW = 890 g-eq/mol was used to obtain composite membranes with diamonds. Finally, films with a ~50 µm thickness and a diamond content of 0.25–5.0 wt. % were obtained. It should be noted that all of the membranes on the base of the Aquivion^®^-type copolymer were annealed at 150 °C; this provided a final structure with an equilibrium arrangement of ion channels and adjacent polymers chains. The surface of membranes tested using atomic force and scanning electron microscopy (AFM and SEM, respectively) showed typical patterns for the pristine membrane and the inclusion of diamond particles in the composite membranes (Figure S2).

Here, we have demonstrated only a few characteristic illustrations (Figures S1 and S2) for the diamonds and membranes; meanwhile, during the preparation of components (copolymers and diamonds) for the initial and composite membranes, as well as the subsequent studies of the samples, we used a number of physicochemical and structural methods, including FTIR, powder X-ray diffraction, dynamic light scattering, viscometry, neutron scattering, electronic (TEM and SEM) and atomic force microscopy, measurements of mechanical properties (Figure S3, Table S1), conductivity, and degree of water absorption for the membranes. The application of these methods has been discussed in detail in a number of recent publications [5,11,13,14,15,16,17,31,32,36,41,42]. In this work, the authors focused on searching for correlations between the structure and conductive properties and the ability of membranes to adsorb water; this predetermined the methods used.

### 2.2. Methods

The proton conductivity of the membrane films was measured through impedance spectroscopy at the maximum equilibrium moistening as a result of boiling in water (100 °C, 1 h). We used a Z-3000X device (Elins, Moscow, Russia) with a 4-electrode circuit for connecting a measuring cell (frequency range 10–150,000 Hz), which contained a moistened sample that was placed and kept at a temperature in the range of 20 to 50 °C. 

The water content in the membrane (W) was determined by comparing the masses (M_WS_, M_DR_) of the samples, which were saturated with water and dried in vacuum to a constant weight at 80 °C, according to the formula W = (M_WS_ − M_DR_)/M_DR_ × 100%. 

The structure of dry films of the Aquivion^®^-type copolymer and composites with diamonds was studied using small-angle neutron scattering (SANS) (YuMO spectrometer, IBR-2 reactor, JINR, Dubna) [45,46,47]. In the SANS experiments (20 °C), the distributions of intensities of neutrons scattered from the samples over the angles (θ) were measured, which corresponded to the range of momentum transfer q = (4π/λ)sin(θ/2) = 0.05–6.0 nm^−1^ for the integral spectrum of neutrons incident on the sample with wavelengths of λ~0.05–0.8 nm [46].

To calibrate the measured spectral intensities, we used a standard sample of vanadium, which scattered neutrons incoherently. The primary processing of the measured spectra with background subtraction and normalization to the data for the standard was carried out using the SAS software package (http://yumo.jinr.ru/software/sas, 19 October 2023) [47]. Finally, we obtained the scattering cross sections of the samples σ(q) = dΣ(q)/dΩ in absolute units per unit solid angle and per cm^3^ of the sample volume vs. momentum transfer (q). Further, we used the general methodology for processing SANS data and physical modeling of the objects under study, taking into account the contrast factors in neutron scattering [48,49].

## 3. Results and Discussions

### 3.1. Conductivity of Membranes with Diamonds

**Figure 2:**
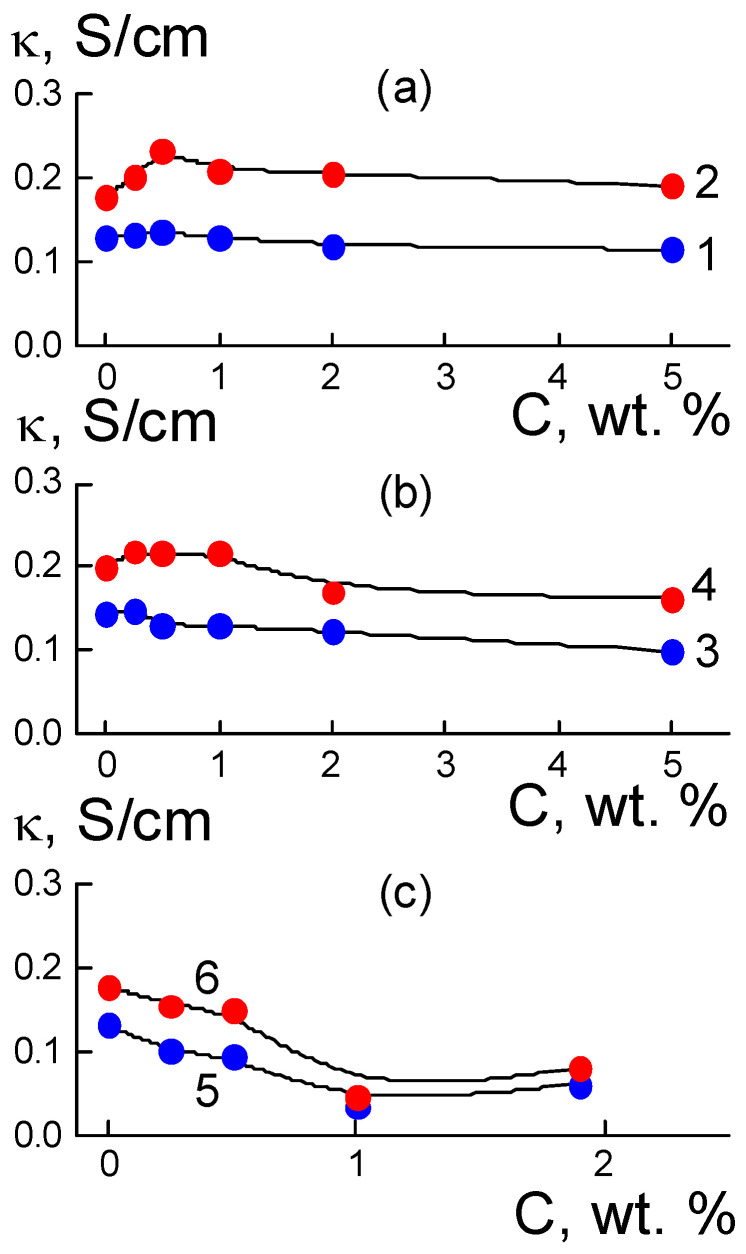
Conductivity data κ(C) for the membranes with DND Z+, DND Z−, or DND-F diamonds (**a**–**c**) vs. filler concentration (C) at temperatures of 20 °C (1,3,5) and 50 °C (2,4,6).

For three series of membranes with DND Z+, DND Z−, and DND-F diamonds, we measured the proton conductivities dependent on the modifier concentration at temperatures of 20 and 50 °C (Figure 2). 

A common feature for all of the samples was the increase in conductivity due to material heating; hydrophilic DND Z+ and DND Z− diamonds stimulated proton transport, especially at low concentrations, C ≤ 1 wt. %, while hydrophobic fluorinated diamonds did not affect conductivity gain at concentrations C ≤ 0.5 wt. %, but caused a diminishing of the thermal effect at C ≥ 1 wt. % together with strong conductivity damping (Figure 2). Further, we normalized the conductivities for the composites κ(C) on similar characteristic κ_0_ for the pristine copolymer membranes without fillers and mainly analyzed the data of κ_N_(C) = κ(C)/κ_0_.

**Figure 3:**
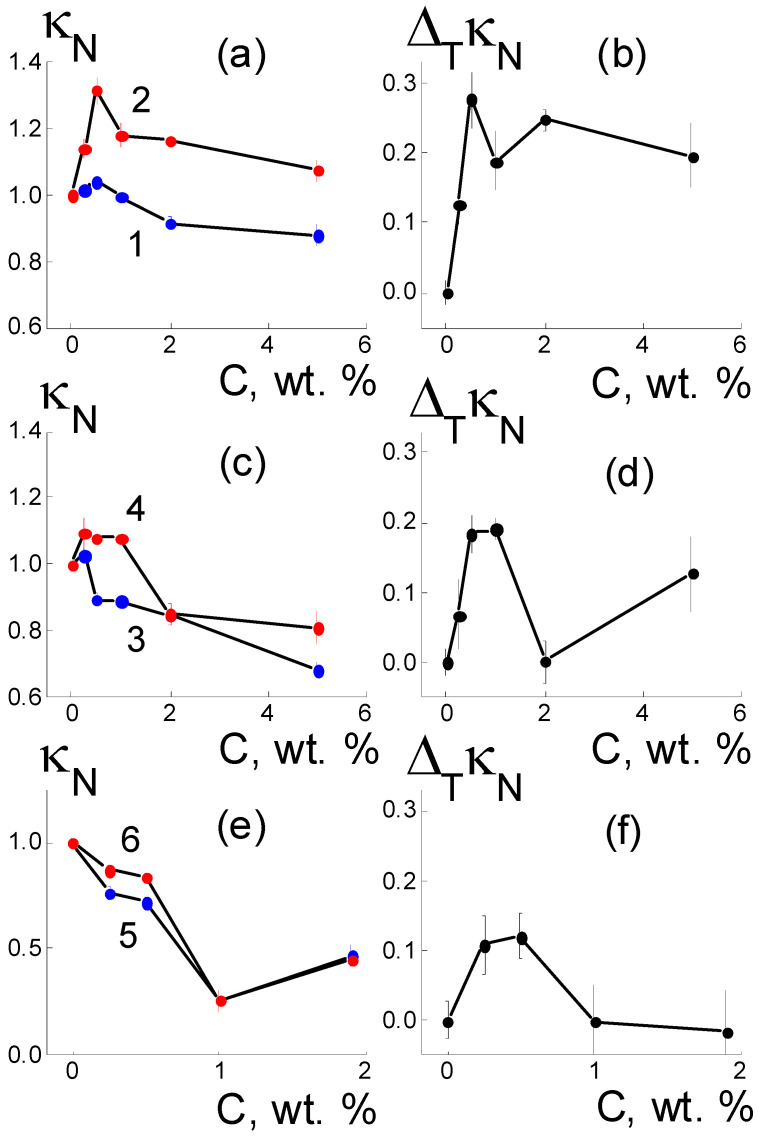
Specific conductivity of membranes κ_N_(C) normalized to the data for the initial copolymer vs. the amount of added DND Z+, DND Z−, and DND-F diamonds (**a**,**c**,**e**) at 20 °C (1,3,5) and 50 °C (2,4,6), as well as corresponding temperature changes in conductivity Δ_T_κ_N_(C) (**b**,**d**,**f**).

As a result of the introduction of detonation DND Z+ diamonds with hydrogen atoms and hydroxyls grafted to the surface into the Aquivion^®^-type copolymer, the composites in the water-saturated state, when heated to a temperature of 50 °C, demonstrated a specific conductivity κ(T) higher than that of the initial matrix, with an increase of up to 30% at a low content of filler C = 0.5 wt. % (Figure 2a and Figure 3a). At the same time (T = 20 °C) the initial effect was only 5% at this fraction of diamonds, but further enrichment of the samples with diamonds caused a decrease in conductivity to ~90% relative to that for the initial copolymer κ_0_ = 0.131 ± 0.002 S/cm (20 °C) (Figure 3a). Above a concentration of C = 0.25 wt. %, the DND Z+ modifier provides a rather stable temperature effect Δ_T_κ_N_(C) = κ_N_(C, 50 °C) − κ_N_(C, 20 °C) ~20–30%. Hence, DND Z+ diamonds are effective activators of proton conductivity in membranes at elevated temperatures (Figure 3a,b).

Compared with DND Z+ particles, DND Z− diamonds with grafted carboxyl groups with a negative charge in an aqueous environment caused a smaller gain in conductivity (~10%) after heating the membrane to 50 °C and conductivity was only stimulated at low fractions in the membranes (0.25–1.0 wt. %) (Figure 3c). Greater amounts (2; 5 wt. %) provoked a decrease in conductivity by ~15% (Figure 2b and Figure 3c). At the initial temperature (20 °C), a modification of membranes with DND Z− particles (0.25–5.0 wt. %) decreased conductivity to ~70% of its value in pristine membranes (Figure 3c). Meanwhile, a temperature effect Δ_T_κ_N_(C) = κ_N_(C, 50 °C) − κ_N_(C, 20 °C) ~20% was comparable to composites with DND Z+, but only at small fractions of DND Z− (0.5; 1.0 wt. %), and it decreased with greater amounts of diamonds (Figure 3d).

It can be seen from the results (Figure 3) that DND Z- diamonds with COOH groups on the surface and at fractions above 1 wt. % reduced the conductivity of the membranes. This can be explained by a violation in the connectivity of the network of ion channels in the copolymers due to the tendency of such particles to segregate at the boundaries of the hydrophilic and hydrophobic zones of the membranes. This trend is not pronounced in membranes with positively charged DND Z+ diamonds, which are attracted to negatively charged sulfonic acid groups of the copolymer; this factor weakens diamond segregation. With different effects of DND Z+ and DND Z- diamonds on the membrane conductivity, the observed effects are of a common nature and are due to the interaction of the hydrophilic filler and polar fragments of the copolymer. 

An alternative variant for the dominance of hydrophobic interactions of components was implemented using fluorinated DND-F diamonds, for which the surface chemical nature was similar to that of the fluorocarbon chains. At a result, this modifier action became stronger by an order of magnitude than that for the hydrophilic DND Z+ and DND Z− diamonds (Figure 2 and Figure 3). In the Aquivion^®^-type copolymer, even a small fraction of DND-F diamonds (1 wt. %) induced a 4-fold decrease in conductivity at both the initial (20 °C) and elevated temperatures (50 °C) (Figure 2c). DND-F particles with a size of ~4–5 nm manifested as large defects in the matrix, which not only perturbed channel packing, but also caused fragmentation. Here, the main role was played by the hydrophobicity of DND-F particles; their presence made it difficult to saturate the membrane with water and prevented proton transport. Heating led to a slight increase in conductivity, Δ_T_κ_N_(C) = κ_N_(C, 50 °C) − κ_N_ (C, 20 °C) ~ 10%; the effect only disappeared with small fractions of diamonds DND-F (0.25; 0.5 wt. %) and at higher amounts (1–2 wt. %) (Figure 3e,f).

**Figure 4:**
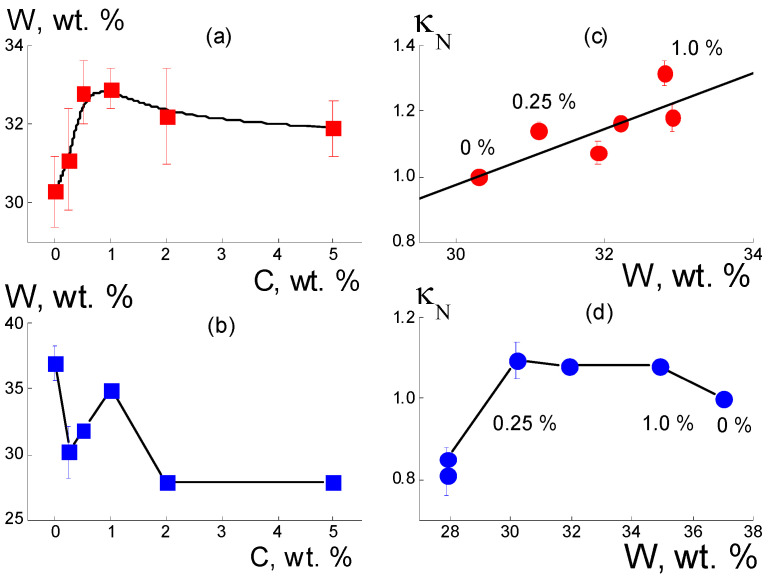
Water saturation W(C) of Aquivion^®^-type membranes vs. the fraction of DND Z+ and DND Z− diamonds (**a**,**b**) in the membranes. Relative conductivity κ_N_(C) of the samples at 50 °C (**c**,**d**) as a function of water saturation W; for the characteristic points, the fractions of diamonds are displayed.

The results (Figure 2 and Figure 3) show the key role played by the diamond functionalization type in the formation of conductive properties of composites based on the Aquivion^®^-type copolymer, as the state of the diamond surface (hydrophilic or hydrophobic) modulated the ability of the membrane to accumulate water. Under conditions of saturation with water, the relative changes in mass (W) and conductivity (50 °C) of composites based on the Aquivion^®^-type copolymer with DND Z+ and DND Z− diamonds are shown in Figure 4. 

In the composites, the increase in the amount of DND Z+ diamonds (C = 0.25–1.0 wt. %) with a water content W(C) ~ 30–33 wt. % reached a maximum; then, at C ≥ 2 wt. %, it decreased slightly (Figure 4a). The step-like profile of the function W(C) reflected the transition from the original to the modified network of water channels with a higher adsorption capacity. This occurred due to the integration of DND Z+ particles into the network, when the area of the hydrophilic inner surface in the membranes became larger due to the diamond–polymer interface.

The introduction of DND Z− diamonds into the membranes in even a small amount (0.25 wt. %) led to a significant reduction (~20%) in the amount of adsorbed water (Figure 4b). Consequently, such diamonds, when interacting with the ionic groups of the copolymer, disrupted the initial structure of the membrane channels. DND Z− particles created closed interfaces (shells) of ionic groups surrounded by non-polar, and this reduced water saturation. With the increase in diamond fraction (0.25 < C ≤ 1.0 wt. %), it overlapping of interfaces became possible. The interfaces opened, allowing water to enter the membranes (Figure 4b). An excess of DND Z− diamonds (C ≥ 2 wt. %) caused segregation in the matrix. This disrupted the channel network, reducing water saturation (Figure 4b). The adsorption properties of composites with DND Z+ and DND Z− particles affected the conductivity of materials differently (Figure 4c,d). For water-saturated composites with DND Z+ particles, a linear correlation of κ_N_ and W values was observed (data at 50 °C, Figure 4c). With a higher water content, the conductivity increased, which indicated the development of a network of water channels via a binding of the copolymer to DND Z+ diamonds (Figure 4c).

**Figure 5:**
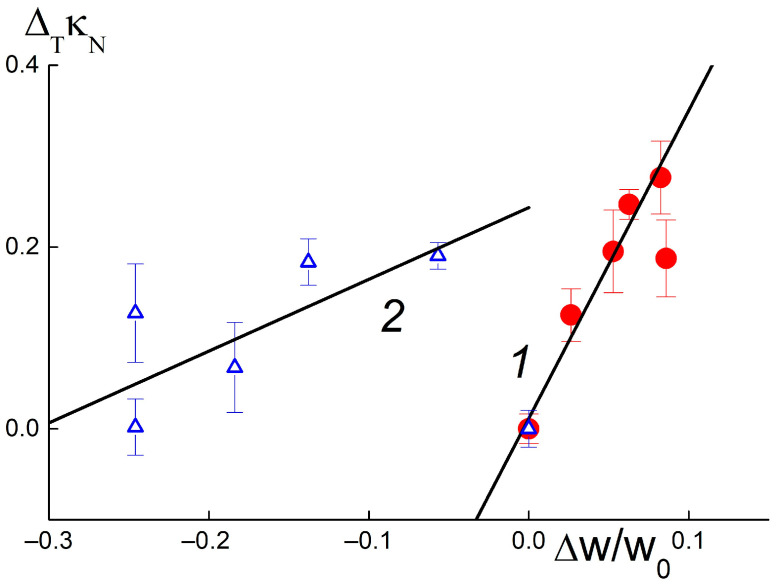
Temperature differences in the normalized conductivity values of composites with DND Z+ (1) and DND Z− (2) diamonds, Δ_T_κ_N_ = κ_N_(C, T = 50 °C) − κ_N_(C, T = 20 °C), depending on the variation in water saturation, ΔW = [W − W_0_]/W_0_, relative to the W_0_ value for the membrane without diamonds.

Under the same conditions, but in the presence of DND Z− particles, greater filling of composites with water W(C) ~ 30–37 wt. % and diamond fractions of 0.25–1.0 wt. % caused sometimes caused a decrease in conductivity (Figure 4d). Consequently, DND Z− particles increased the porosity and water capacity of the membranes, disrupting the network of conductive channels. Despite the differences in electrical and adsorption properties, both composites showed similar temperature changes in conductivity Δ_T_κ_N_ = κ_N_(C,T = 50 °C) − κ_N_(C, T = 20 °C) when varying the water saturation ΔW(C) = [W(C) − W_0_]/W_0_ relative to the W_0_ level for the pure membrane without diamonds (Figure 5). It was established that the differences Δ_T_(κ/κ_0_) depended linearly on the values of ΔW,

Δ_T_κ_N_ = α + β·ΔW. (1)

Parameter α corresponds to the value Δ_T_κ_N_ at ΔW = 0 and coefficient β indicates derivative d[Δ_T_κ_N_]/d(ΔW). For composites with DND Z+ and DND Z− diamonds, the parameters found from the approximation of the data by Function (1) differed greatly:

α = 0.012 ± 0.025; β = 3.39 ± 0.50 (DND Z+);

α = 0.24 ± 0.04; β = 0.79 ± 0.26 (DND Z−).

Compared with DND Z− particles, DND Z+ diamonds stimulated a temperature increase in membrane conductivity that was four times stronger. As a result of the positively charged, hydrogen-saturated surface, the DND Z+ particles acted as effective modifiers, and were embedded into the matrix; this created additional water-filled ion channels and linked them into a conductive network. 

The effect of diamonds on membrane hydration was estimated using a simple model, assuming that DND Z+ diamonds with a diameter of d_p_ = 4.5 nm and a volume of V_1_ = πd_p_^3^/6 create water shells with an outer radius R and thickness δ = (R − d_p_/2) around themselves. In a composite with a diamond content of 1 wt. % and numerical volume concentration of particles N = 1.3 × 10^17^ cm^−3^, the water shells occupied a volume fraction equal to V_t_ = NV_1_[(2R/d_p_)^3^ − 1] = 6.6%, according to the weight gain of the sample when moistened ΔW/W_0_ = 3% (Figure 4a), taking into account the density of the copolymer (2.2 g/cm^3^). Using the equation for V_t_(R), we found the radius R = 5.1 nm and the thickness of the water shell around the particle δ = (R − d_p_/2) = 2.8 nm. The latter was comparable to the diameter of ion channels in moistened membranes of perfluorinated copolymers [5]. Thus, each diamond particle formed the surrounding water volume V_wd_ ≈ V_1_[(2R/d_p_)^3^ − 1] ≈ 10V_1_ at an order of magnitude greater than its own. Therefore, DND Z+ diamonds with a content of 1 wt. % enhanced matrix hydration by ~10% and provided a comparable gain in conductivity (Figure 4c).

### 3.2. Membrane Structures 

#### 3.2.1. Composites with DND Z+ Diamonds

The mechanism of effect of DND Z+ on the conductivity of composites based on the Aquivion^®^-type copolymer was elucidated from the analysis of the structural changes in the membranes. In dry composites, with the increase in the amount of diamonds (0.25; 0.5; 1.0; 2.0; 5.0 wt. %), we observed progressive growth in SANS cross section σ(q) at momenta q ~ 0.01–1.0 nm^−1^ according to the power-law of σ(q,C) ~ q^−2^, which is characteristic of Gaussian polymer chains and chain structures of the particles observed in aqueous diamond dispersions [14,15].

**Figure 6:**
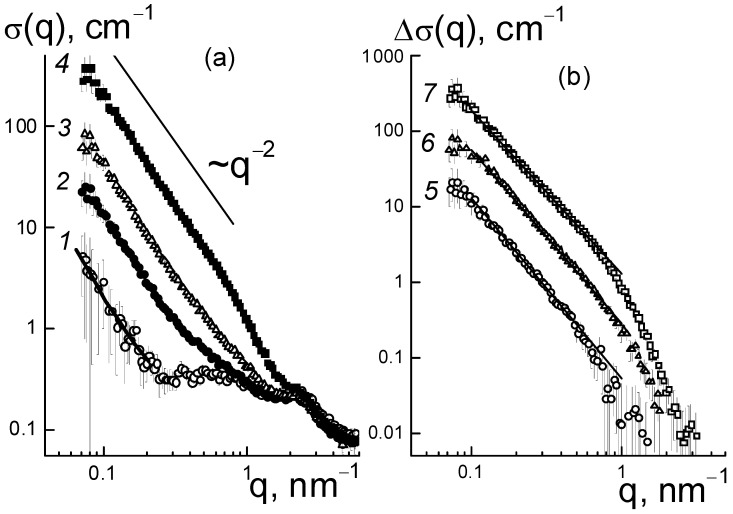
SANS cross sections σ(q,C) (**a**) in membranes with various DND Z+ contents (C = 0; 0.25; 1.0; 5.0 wt. %) (1–4) and differential data Δσ(q,C) (C = 0.25; 1.0; 5.0 wt. %) (5–7) relative to the pure membrane (**b**), dependent on momentum transfer. The line for composites denotes the fractal behavior of the cross sections σ(q,C) ~ q^−2^. For the pure copolymer (1), the fractal approximation function (**a**) is constructed. Differential cross sections (**b**) are approximated by fractal scattering functions (lines) in a limited range of momentum values.

This is illustrated in Figure 6a for the samples with several characteristic modifier concentrations. In this case, because of the high contrast against the copolymer, the diamonds in the membranes, even at a low content (0.25 wt. %), caused scattering of almost an order of magnitude higher than in the original copolymer (Figure 6a). When increasing the fraction of diamonds up to 5.0 wt. % the scattering raised by two orders of magnitude at a low momenta q ≤ 0.1 nm^−1^, while the cross section profiles remained similar (Figure 6a). Under conditions of intense scattering from diamonds relative to the copolymer, the cross sections associated with them in the first approximation were found through a subtraction of the copolymer contribution, Δσ(q, C) = σ(q, C) − σ(q, C = 0) (Figure 6b).

In the samples, the data showed fractal behavior, Δσ(q,C) ~ q^−Df^ (Figure 6b) with the index D_f_(C) ~ 2, which indicated diamond binding into chain aggregates. Similar structures are usually observed in aqueous dispersions of diamonds [14,15]. In our case, diamond aggregates formed within the mixed dispersion of diamonds and a copolymer in dimethylformamide (DMF), and were preserved during precipitation of components, removal of the solvent, and heat treatment of the resulting membrane films to achieve an equilibrium structure in the SO_3_H form.

**Figure 7:**
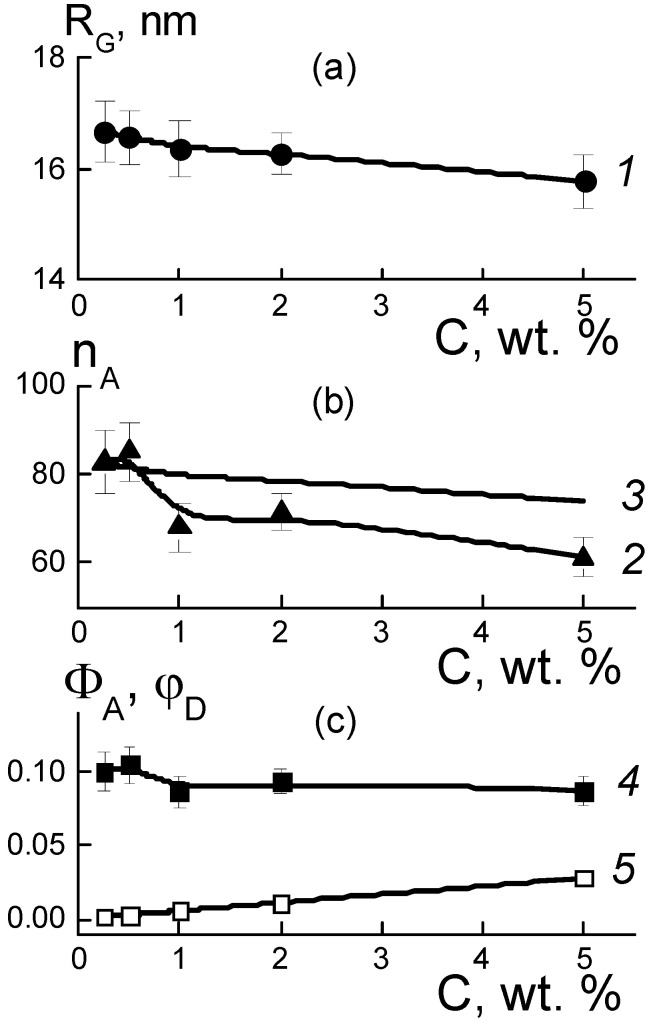
Concentration dependences of the gyration radius R_G_(C) (1), the measured (2) and calculated (3) aggregation numbers n_A_(C), the volume fractions Φ_A_(C) of diamonds DND Z+ in aggregates (4), and average value of Φ_A_(C) in membranes φ_D_(C) (5) (**a**–**c**).

Before a detailed analysis of the fractal nature of the organization of diamonds in the composites, the size of the diamond aggregates was estimated from an approximation of the data at a low momentum using the Guinier function,

Δσ(q,C) = I_o_exp[−(qR_G_)^2^/3], (2)

where R_G_(C) is the gyration radius of observed objects, I_o_(C) = ΔK_DP_^2^φ_D_v_P_n_A_ is their cross section in the limit q → 0 dependent on the contrast factor of diamonds against the polymer (ΔK_DP_), their volume fraction φD, the volume of a particle (v_P_), and the aggregation number (n_A_). The radii R_G_(C) and the numbers n_A_(C) = I_o_(C)/ΔK_DP_^2^φ_D_v_P_ found from the I_o_(C) data are shown in Figure 7a,b.

These parameters made it possible to determine the volume fractions of particles Φ_A_ = n_A_v_P_/V_A_ (Figure 7c) in aggregates with a volume of V_A_ = (4π/3)^3/2^R_G_^3^ [14]. It was found that by increasing the amount of modifier (C = 0.25–5.0 wt. %), the volume fraction of diamonds in the aggregates Φ_A_(C) ~ 9–10% remained almost constant and was three times higher than the average level (~2.8%) for the sample in the upper limit of concentrations (5 wt. %) (Figure 7c). Thus, diamonds in the copolymer matrix created a density-stable nano-sized phase similar to that of diamond hydrogels [15].

Taking into account the fractal behavior of the sections ~q^−2^ (Figure 6b), we considered the correlation between the parameters n_A_(C), R_G_(C) as in a Gaussian chain of diamonds. Then, the aggregation numbers n_GA_ = 6(R_G_/d_P_)^2^ were defined by the ratios of gyration radii to diamond size (d_P_) (Figure 7b, data 3). These n_GA_ magnitudes agreed with the experiment at low diamond fractions C = 0.25–0.5 wt. %, when scattering by individual aggregates predominated. 

At C = 0.5 wt. %, the diamond amount N_DND_ = 6.0 × 10^16^ cm^−3^, the n_A_ ≈ 80 (Figure 7b) and the number of aggregates N_AG_ = N_DND_/n_A_ = 7.1 × 10^14^ cm^−3^ defined their diameter L_GA_ = √6 R_G_ ≈ 40 nm, which was three times lower than the average spacing between them R_INA_ = (N_DND_/n_A_)^−1/3^ ≈ 110 nm. Hence, an overlapping of aggregates was unlikely. This was also favored by the structure of the Aquivion^®^-type copolymer matrix [11] composed of domains with a radius of ~100 nm. With domains of this size, it is realistic that aggregates can be filled in the gaps between them.

**Figure 8:**
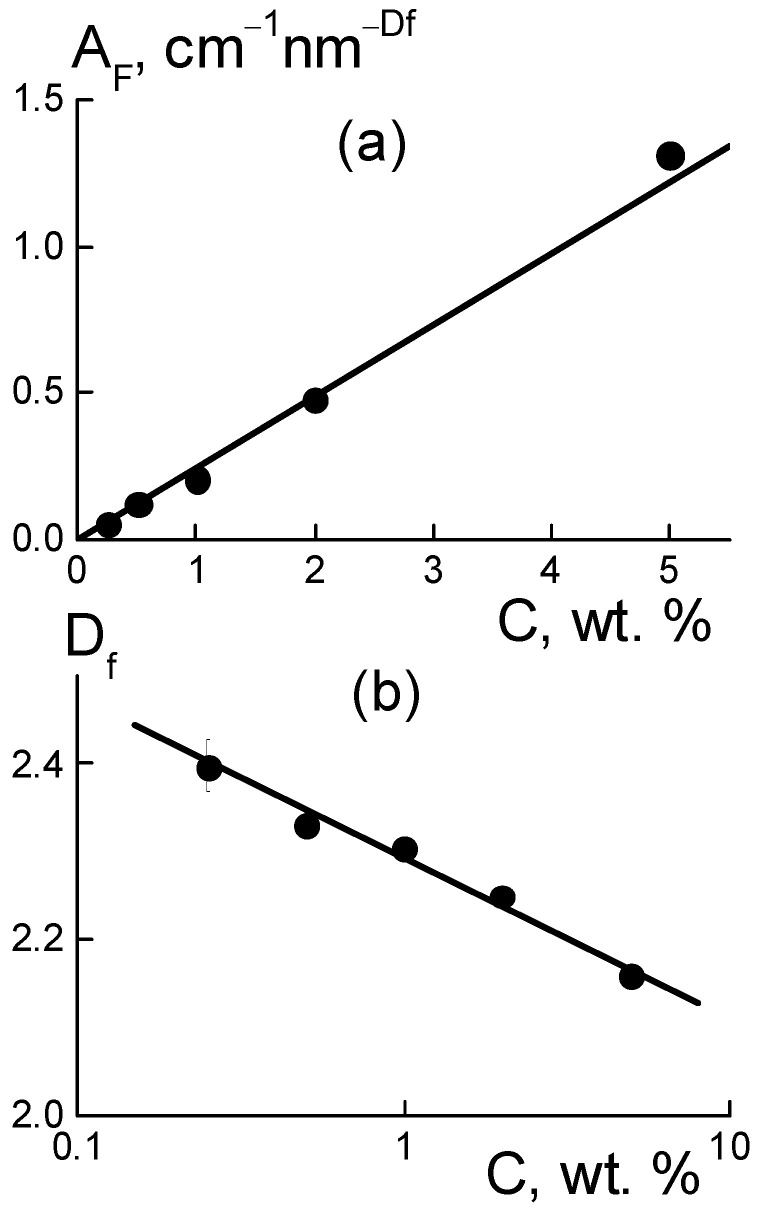
Parameters of approximation of cross sections Δσ(q,C) using the fractal function at various proportions of fillers in the composites: (**a**) coefficient A_F_(C) and (**b**) fractal dimension D_f_(C) of diamond structures.

Meanwhile, in the composites with a high filler content, C = 5 wt. %, the numbers of diamonds and aggregates, N_DND_ = 6.0 × 10^17^ cm^−3^ and N_AG_ = 9.8x10^15^ cm^−3^, were larger by an order of magnitude. On average, the aggregates were spaced at a distance R_INA_ = N_AG_^−1/3^ ≈ 50 nm approaching a diameter of L_GA_ ≈ 40 nm. So, it seems their interaction is inevitable, displaying with some trends towards forming bigger and more substantial structures. However, the observed lowering of parameters R_G_(C), n_A_(C) (Figure 7a,b) indicated no progress in aggregation, but instead a dominating binding of diamonds with the ionic copolymer groups. This trend was also manifested in the fractal geometry of the chains that formed the aggregates (Figure 8).

In the range q ~ 0.1–0.6 nm^−1^, the differential cross sections (Figure 6b) obeyed a fractal dependence Δσ(q,C) = A_F_q^−Df^ with proportionality parameter A_F_(C) and index D_f_(C) ~ 2.2–2.4 (Figure 8b), which indicated some branched chain structures. The parameter A_F_(C) = α_F_·C characterized the chain scattering power and, thus, was proportional to the concentration of diamonds with a factor α_F_ = 0.24 ± 0.01 cm^−1^ nm^−Df^ (Figure 8a).

The meaning of the parameter A_F_ became clear when using the model scattering function for the chains, [1 + (qR_C_)^2^]^−Df/2^ ≈ 1/(qR_C_)^Df^, at momenta q >> 1/R_C_ above the reciprocal correlation radius of the chain R_C_. Such a scattering function corresponded to a chain with the aggregation number n_C_ = (R_C_/r_C_)^Df^, given by the ratio of R_C_ to the correlation radius r_C_ of particles forming a chain with the fractal dimension D_f_. Then, the coefficient, α_F_ = (0.01)(ρ_P_/ρ_D_)(ΔK_DP_)^2^v_P_/r_C_^Df^, is a function of r_C_ variable and the following parameters are known: the contrast factor between the diamonds and polymer (ΔK_DP_), the volume fraction of diamonds (φ), particle volume (v_P_), and polymer and diamond densities (ρ_P_, ρ_D_). Using the α_F_ value, we found r_C_ ≈ 2.2–2.3 nm, which was comparable to a similar parameter of r_C_ = (2/3)d_P_ ≈ 3 nm for a diamond. Finally, this confirmed the validity of the chain model for diamond assembly. 

It is notable that the fractal dimension of the chains decreased logarithmically with the increase in diamond fraction, D_f_(C) = D_1_ − β_f_ ln(C), with parameters D_1_ = 2.29 ± 0.01, β_f_ = 0.079 ± 0.006, where D_1_ corresponds to the content of diamonds C_1_ = 1 wt. %. Such a decrease in the D_f_(C) dimension (Figure 8b) resulted in a transition to less branched structures, in accordance with the weakening of aggregation when the membranes were enriched with a modifier (Figure 7b) due to progressive binding of the hydrophilic diamonds to the ionic groups of the polymer.

The observed transformation of diamond structures associated with the formation of diamond−polymer interfaces should have affected the network of ion channels in the matrix. However, the scattering patterns showed a relatively stable position for the ionomer peak (q* ~ 2 nm^−1^) (Figure 6a). Hence, the DND Z+ diamonds were compatible with the copolymer and did not interfere with the formation of its inherent supramolecular structure with hydrophobic domains and ion channels during a packing period L_C_ ~ 2π/q* ~ 3 nm. Meanwhile, the diamonds modulated the characteristics of ion channels and affected their ordering in the polymer matrix.

**Figure 9:**
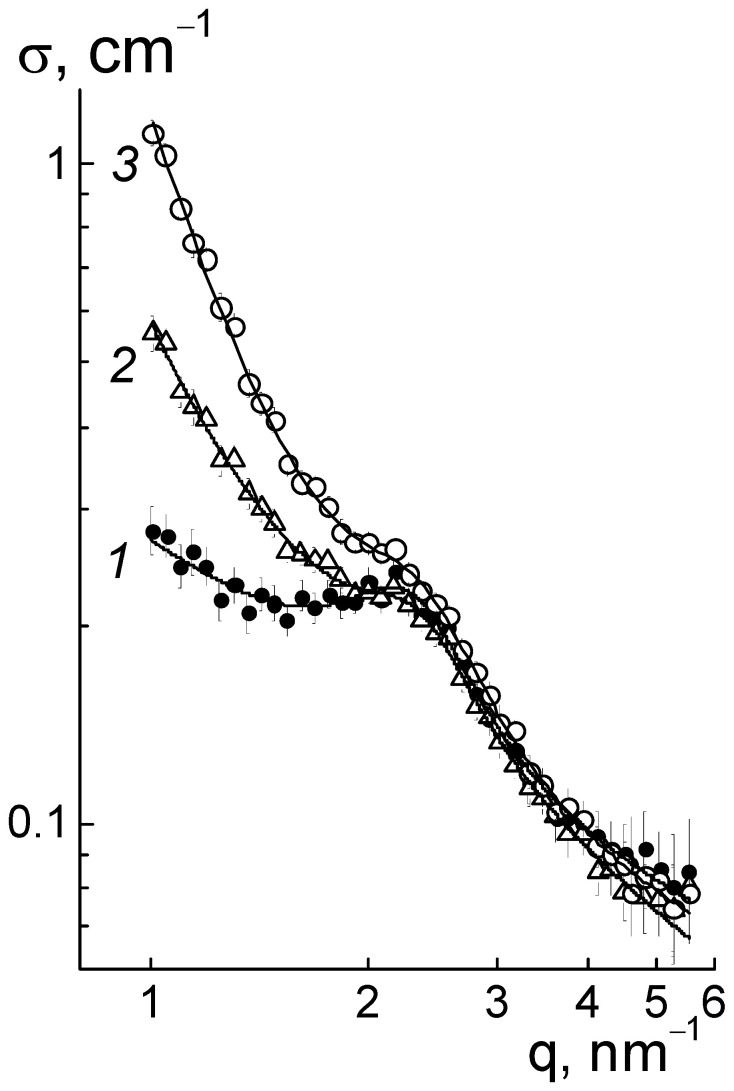
Ionomer peak approximation with Function (3): 1—pristine membrane; 2—composites with 2.0 (2) and 5.0 wt. % (3) for the DND Z+ diamonds.

**Figure 10:**
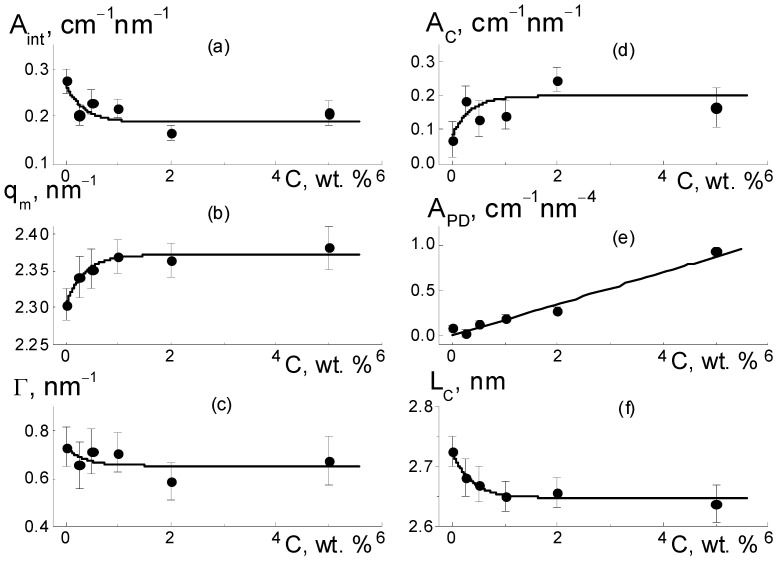
Structural parameters of the composites depending on the fraction (C) of DND Z+ diamonds: (**a**–**c**) amplitude A_int_(C), position of the maximum q_m_(C), width Γ(C) of the ionomer peak; (**d**,**e**) factors A_C_(C), A_PD_(C), characterizing scattering from linear fragments of channels and diamond-polymer interfaces; and (**f**) channel packing period L_C_(C). Curves (**a**–**d**,**f**) show the approximations with Function (4). For A_PD_(C) data, a linear fit is plotted (**e**).

For the composites, the behaviors of cross sections (Figure 6a) at momenta q ≥ 1 nm^−1^ were analyzed using the following function (Figure 9),

σ(q,) = (A_int_/q)[1 + (q − q_m_)^2^/Γ^2^]^−1^ + A_C_/q + A_PD_/q^4^ + B_g_, (3)

which included the contributions of the ionomer peak (Lorentzian with amplitude A_int_, maximum position q_m_, half-width at half-maximum Γ), linear fragments of channels (~1/q), diamond surface (~1/q^4^), factors A_C_, A_P_), and background additive (B_g_). For all of the membranes, the sections obeyed Function (3), which is shown for several samples (Figure 9). The approximation parameters are given in Figure 10.

As they are dependent on diamond concentration (Figure 10), all the parameters (except for the diamond-related A_PD_ factor) follow a common law

P(C) = P_0_ + ΔP[1 − exp(−C/C*)] (4)

where P_0_ = P(C = 0), and ΔP is the initial parameter and its variation at filler content C >> C* = 0.35 ± 0.10 wt. % is far above the critical value found from the approximation of q_m_(C) data using Function (4) (Figure 10b). 

As a result of the presence of diamonds, the copolymer structure relaxed from a pristine to modified state when the diamond fraction exceeded a certain level (C*), at which the distance between DND Z+ particles, on average, decreased to ~30 nm. This was comparable to the diameter of bundles (fibrils) composed of ion channels in perfluorinated copolymers [18,19,20,21,22,23,24,25,26,27,28,29,30]. Further membrane filling (C > C*) meant the integration of particles into a network of ion channels.

According to the data (Figure 10a), due to the copolymer with DND Z+ modification, the amplitude of the ionomer peak decreased, ΔA_int_(C)/A_int_(C = 0) ~ 30%. However, the peak width (Γ) remained practically unchanged (Figure 10c). Consequently, the diamonds did not disturb the coordination of channels in bundles with a transverse size L_coh_ = 2π/Γ ~ 10 nm (scattering coherence length). The observed shift in the ionomer peak (q_m_) (Figure 10b) demonstrated a reduction in the period L_C_ = 2π/q_m_ ~ 3 nm of channel packing, ΔL_C_/L_C_ ~ 3% (Figure 10f). This indicated channel compression due to electrostatic attraction between the ionic groups of the diamonds and copolymers. As a result, the interference effect (A_int_) was reduced (Figure 10a).

**Figure 11:**
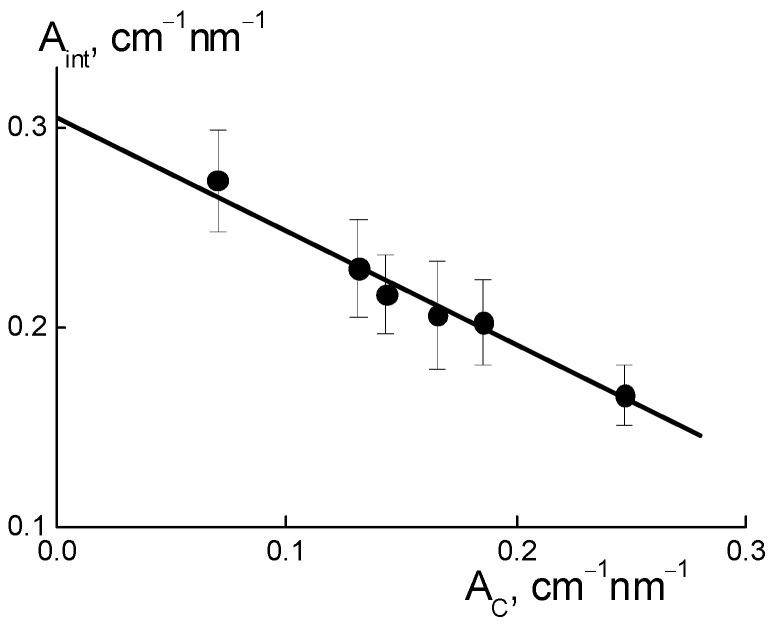
Linear decrease in the amplitude A_int_(C) of the interference peak with an increase in the factor A_C_(C) separately characterizing scattering on the channels.

Such interference (A_int_) weakening (Figure 10a) was caused by the formation of diamond-polymer interface enclosed ionic groups for both components. Such a process meant building additional ion channels, which is evident from the increase in the total scattering ability of channels (A_C_) (Figure 10d). Along with this, we discovered a linear correlation between the parameters (Figure 11),

A_int_(C) = A_I_ − B_I_A_C_(C); (5)

A_I_ = 0.306 ± 0.007 cm^−1^ nm^−1^, B_I_ = 0.57 ± 0.04.

The excess in scattering from channels (A_C_), together with interference damping (A_int_) (Figure 11), should be treated as a result of diamond-polymer interface formation when increased ionic groups in the copolymers do not create their own channels. A lowered interference effect from channel packs (bundles) was not compensated for by scattering from the interface, with different scattering abilities than that for the ionic channels in the matrix. The observed correlation between parameters A_int_(C) and A_C_(C) can be explained by a linear increase in the values A_C_(C) = A_C0_ + β_D_C with the concentration of diamonds. They created additional ion channels with a certain ability (factor β_D_) through the integration of ionic copolymer groups. As a result, the quantity of polymer channels also decreased linearly.

As a result of the decrease in total volume of polymer channels, the amplitude of the ionomer peak was weakened, A_int_(C) = (ν_C_ − 1)(A_C0_ − α_IG_·C), where ν_C_ is the number of channels collected in a bundle and α_IG_ is the coefficient that determines the rate of decrease in the volume fraction of polymer channels. This implies a linear relationship between the parameters A_int_(C) and A_C_(C) (Figure 11),

A_int_(C) = (ν_C_ − 1)A_C0_(1 + α_IG_/β_D_) − (ν_C_ − 1) (α_IG_/β_D_)A_C_(C). (6)

From Equation (6) with the A_I_ = ν_C_A_C0_(1 + α_IG_/β_D_), B_I_ = ν_C_(α_I_/β_D_) parameters found above, we computed the ratio (α_IG_/β_D_) ≈ 0.15 and channel number in a bundle ν_C_ = B_I_/(α_I_/β_D_) + 1 ≈ 5. A low magnitude of (α_IG_/β_D_) ≈ 0.15, the formation of hybrid channels was shown through the diamond-polymer interface. This was seven times more active than the growth of the polymer channel’s lack. Owing to the substitution of polymeric ion channels by hybrid planar ones, there was a good profit in the water adsorption and proton conductivity in the membranes when modified with DND Z+ (Figure 4a,b).

With the concentration of diamonds (Figure 10e), the interface area (S_DP_) increased linearly according to the dependence A_PD_ = 2π(ΔK_DP_)^2^S_DP_φ_F_ = k_P_·C, where the coefficient k_P_ = 0.17 ±0.01 cm^−1^ nm^−4^ is proportional to the fraction φ_F_ of free surface in the total area of the diamond interface. Using the k_P_ value, we estimated the magnitude of φ_F_ ≈ 2/3. We found that a third of the diamond surface was not manifested in scattering due to joint crystal facets in chains when the fraction of the open surface was almost the same as for the contacts of the cubic particles.

In general, our analysis showed that in the copolymer matrix, when modified with diamonds (C = 0.25–5.0 wt. %), the channel bundles were retained like in the pure copolymer, and these packages with a transverse size of ~10 nm were composed of ~5 channels. However, in the composites, the channel packing density increased by ~6% as a result of a reduction in the spacing between channels, L_C_ ~ 2.6–2.7 nm. 

**Figure 12:**
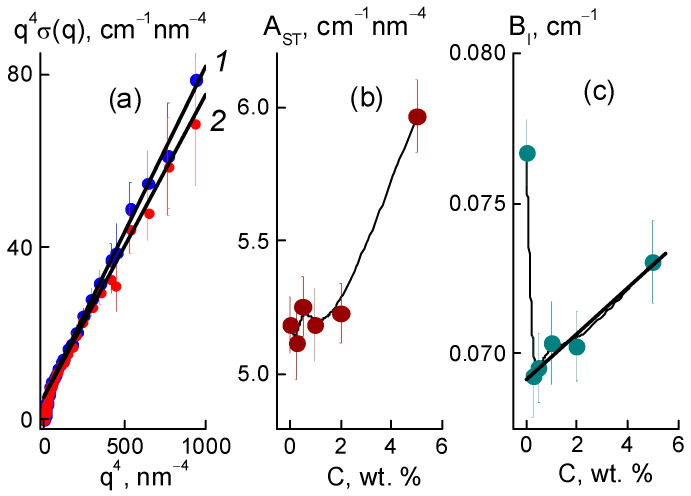
Modified cross sections q^4^σ(q) vs. q^4^ for membranes with DND Z+ contents C = 0; 1 wt. % (1, 2), approximation using Equation (7) is shown (**a**). A_ST_(C) and B_I_(C) (**b**,**c**) parameters for different fractions of diamonds (C), linear approximation for B_I_(C) data is presented (**b**).

Further, we found a narrowing of ion channels from the estimates of their total area. Direct information about the surface of the ion channels in the membranes was obtained from SANS at a high momenta q ≥ 3 nm^−1^, when channel boundaries and diamond facets predominantly induced scattering according to Porod’s law, σ(q) ~ q^−4^. For the samples, the modified sections q^4^σ(q) dependent on the argument q^4^ demonstrated linear behaviors (Figure 12a),

q^4^σ(q) = A_ST_ + B_I_·q^4^, (7)

where parameter A_ST_ = 2π[(ΔK_P_^2^)S_TP_ + (ΔK_DP_^2^)S_TD_] includes the contributions proportional to the areas S_TP_, S_TD_ of the polymer channel surface, and the diamond-polymer interface. The A_ST_ depends on the contrast factors for channel walls and diamond-polymer interfaces, ΔK_P_ = 4.3 × 10^10^ cm^−2^ and ΔK_DP_ = K_D_ − K_P_ = 7.4 × 10^10^ cm^−2^, where the scattering length densities for the diamond and polymer were K_D_ = 11.7 × 10^10^ cm^−2^, K_P_ = 4.3 × 10^10^ cm^−2^, respectively. The B_I_ coefficient is the cross section for scattering from individual atoms in the membranes. A_ST_, B_I_ are plotted vs. the diamond fraction in the membranes (Figure 12b,c).

Filling with diamonds up to 5 wt. % increased the area of internal boundaries in the membranes according to the growth of the A_ST_ parameter (Figure 12b). Here, the increase ΔA_ST_ ≈ 0.8 cm^−1^ nm^−4^ corresponded approximately to the gain in similar characteristic A_PD_(C) found for the diamonds at momenta q ≥ 1 nm^−1^ (Figure 10e). Note, A_ST_(C) included contributions from both the polymer and diamonds (Figure 12b). Therefore, A_ST_(C) deviation from a linear dependence was associated with a reduction in the polymer channel surface in the composites.

Based on the value of A_ST_ for the pure copolymer, we found the integral area of channels S_TP_ = A_ST_/2π[(ΔK_P_^2^) ≈ 4.5 × 10^6^ cm^2^/cm^3^. Because of the large number of polymer ionic groups, N_GR_ = 1.35 × 10^21^ cm^−3^, which created channels, area S_TP_ was greater by an order of magnitude than the one for the free borders of diamonds S_tD_ = 3.9·10^6^ cm^2^/cm^3^, even at a diamond content of 5.0 wt. %. In the polymer channel, there was a small area per a group, S_1_ = S_TP_/N_GR_ ≈ 0.3 nm^2^. On average, the distance between groups √S1 ≈ 0.5 nm was close to the length of the polymeric side chains with the terminal groups. This ensured their overlap when they covered channel surfaces.

For the pure membrane from the integral area S_TP_ and the packing period of channels L_C_ (Figure 10f), we estimated their diameter, δ_C_ ≈ S_TP_L_C_^2^/π ≈ 1 nm. Locally, the channels were linear within the distance limited by the size of the polymer domains, L_P_ ~ 2π/q_mp_ ~ 12 nm, in accordance with the position of wide scattering peak q_mp_ ≈ 0.6 nm^−1^, which reflected the packing of domains in the matrix (Figure 6a). This size was consistent with the scale of the coherence region in scattering from channel bundles, L_coh_ = 2π/Γ ~ 10 nm, estimated using the width of the ionomer peak (Figure 10c). In the copolymer matrix, the channel units with a length of ~ L_P_ formed branched structures with a fractal dimension D_cf_ = 2.6 ± 0.3. This parameter was found from an approximation of SANS data in the interval q = 0.07–0.3 nm^–1^ using the function σ(q) = A_cf_/q^Dcf^ + B_cf_ with the parameters A_cf_ = 0.0049 ± 0.0034 cm^−1^ nm^−Dcf^, B_cf_ = 0.17 ± 0.06 cm^−1^ (Figure 6a, data 1).

**Figure 13:**
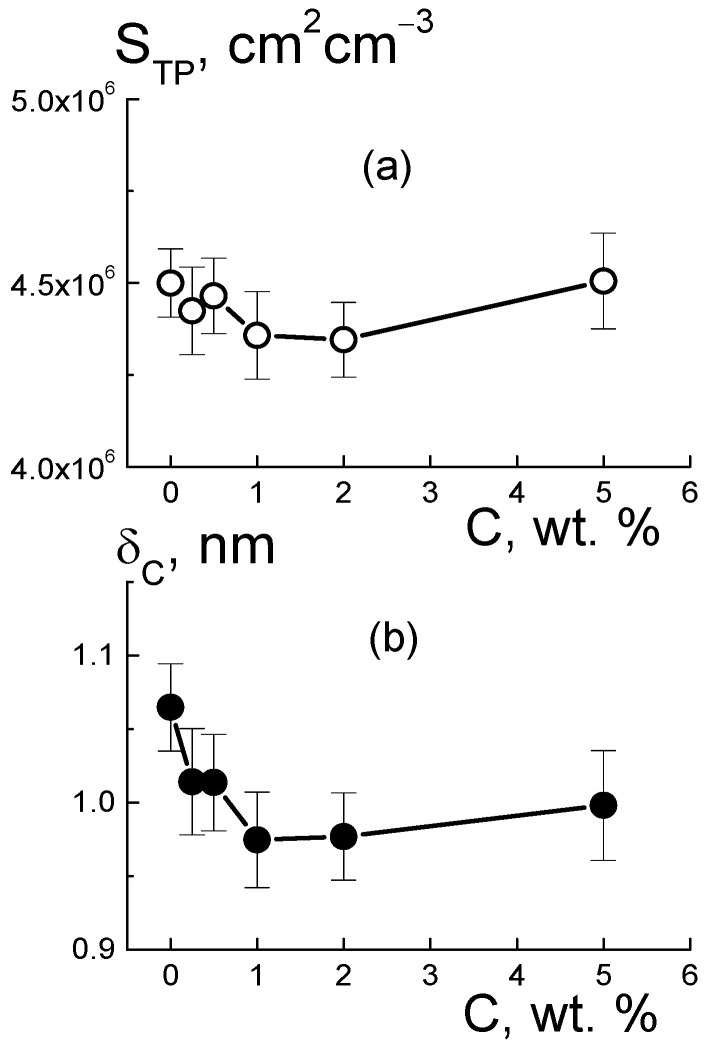
Integral surface area S_TP_(C) (**a**) and diameter of polymer ion channels δ_C_(C) (**b**) vs. fraction of diamonds in membranes.

Then, we determined the characteristics of the polymeric channels in the composites using the A_ST_(C) parameter (Figure 12b) corrected for the contribution of diamond borders, A_PD_(C). In this way, we evaluated the surface area S_TP_(C) = [A_ST_ − A_PD_]/2π(ΔK_P_^2^) and polymer channel diameter δ_C_ = S_TP_L_C_^2^/π in the membranes with various proportions of diamonds (Figure 13). The addition of diamonds up to 1 wt. % into the membranes caused a reduction in the total area of the polymer channels (S_TP_) by ~3% and a narrowing in their diameter (δ_C_) by ~8% (Figure 13). The enrichment of membranes with diamonds (5 wt. %) did not affect the channel diameters (δ_C_ ≈ 1 nm) (Figure 13b), as their area was restored to the initial level (Figure 13a). The behaviors of these characteristics allowed us to state that the increase in total channel length was ~10%. This effect could be attributed to the partial segregation of excess diamond when it disturbed the copolymer structure less.

The data in Figure 13b explain the shortening in the packing period of channels (L_C_) (Figure 10f) as a consequence of the compression of the channels themselves at a stable shell thickness when the copolymer was modified with DND Z+. Despite channel squeezing, the membranes obtained an enhanced ability to absorb water. This was revealed in the behavior of the B_I_(C) parameter (Figure 12c). Although the magnitude of B_I_(C) decreased sharply when a small fraction of diamonds (0.25 wt. %) was introduced into the copolymer, the enrichment of the samples with diamonds caused linear growth of the parameter (Figure 12c). In following analysis, we compared these results with the data for the nuclei of the elements (C, F, O, S, and H) in the samples, taking into account the nuclear coherent lengths b_ci_ (i = 1, 2, …, 5) and the incoherent cross sections σ_INi_/4π,

B_Iest_ = Σ(b_ci_^2^ + σ_INi_/4π)N_i_, (8)

where N_i_ is the numerical concentration of atoms.

Using the data [49] and Equation (8), we calculated B_Iest_ ≈ 0.037 cm^–1^ for the dry copolymer (density ρ = 2.2 g/cm^3^) with the equivalent weight EW = 890 g-eq/mol. The copolymer was composed of fragments -[CF_2_-CF_2_]_n_-[CF_2_-CFG_S_]- consisting of tetrafluoroethylene units (n ≈ 6) and a unit carrying the side group G_S_ (-O-CF_2_-CF_2_-SO_3_H).

The experimental value of B_I_ = 0.077 ± 0.001 cm^−1^ exceeded the estimate, as the sample contained bound water, similar to the usual amount in films of Aquivion^®^-type copolymers from the mechanical tests (~6 wt. %) [50]. The difference B_I_-B_Iest_ = 2(σ_H_/4π)N_W_ ≈ 0.04 cm^−1^ was determined by the proton incoherent scattering cross section, σ_H_ ≈ 80.3 × 10^−24^ cm^2^ [49] and water molecule concentration N_W_. As a result, we found that each SO_3_H group was linked to λ_W_ ≈ 2.3 water molecules on average. This corresponded to water mass and volume fractions of Wm = 4.7 wt. %, Wv = 8.8 vol. %, respectively.

Such treatment of B_I_(C) data for membranes with a variation in diamond fraction showed a significant influence of the DND Z+ filler on the adsorption properties of the membranes. Even a small filler amount (0.25 wt. %) caused a substantial decrease in the parameter, δB_I_ ~ 10% (Figure 12c), and lack of water in the composite, ΔWv = 1.8% (20% reduction). The diamonds, which occupied 0.17% of the membrane volume, could not substitute an amount of water an order of magnitude greater. However, due to electrostatic attraction to the ionic groups of the copolymer, these diamonds could block some of the channels and reduce the degree of hydration of the SO_3_H groups. With further modification, the adsorption capacity of the membranes was restored, as the diamonds with hydrogen atoms and hydroxyls at the surface formed hydrogen bonds with water molecules and the hydrophilic diamond-copolymer interface attached well to water. The estimates below endorse this mechanism. 

The saturation of membranes with diamonds (5 wt. %, volume content of 3.4 vol. %), when their numerical concentration reached a high magnitude, N_DND_ ≈ 6 × 10^17^ cm^−3^, led to linear growth of the B_I_ parameter (Figure 12c). The gain δB_I_~5.5% corresponded to the relative additive water volume ΔWv = 0.84%. If we associate this amount with diamonds, then each DND Z+ particle could accept ~470 water molecules, covering the entire available diamond surface, S_D1_ ≈ πd_P_^2^(2/3) ≈ 42 nm^2^, where the factor (2/3) was a part of the open surface in diamond aggregates, as estimated above from the data in Figure 10e.

In fact [13,35], the number of functional groups (H, OH) grafted to a diamond was an order of magnitude less than the actual amount of water molecules. Hence, high adsorption occurred mainly due to polymeric ionic groups forming hydrophilic layers around the diamonds, and this assumption was confirmed by the conductivity effect (Figure 3a,b). For a particle, we obtained a number of polymeric ionic groups in the interface, n_ig_ = S_D1_/S_1_ ≈ 140, where S_1_ ≈ 0.3 nm^2^ is the area per group on the surface of the channels. The SO_3_H group was attached to approximately 2.3 water molecules and the number per diamond particles was ~320; as a result of diamond group hydration, the total amount of water molecules was ~400, which was in agreement with the experimental quantity (~470).

As stated above, the developed a diamond-copolymer interface stimulated water adsorption (Figure 4a), and the hybrid diamond-polymer channels helped increase conductivity (Figure 4c). In the composite with a high modifier fraction (5 wt. %), the numerical concentration of particles N_DND_ = 6.0 × 10^17^ cm^−3^ presented a minimal gap between the diamond particles, ΔR_D_ ≈ (N_DND_^−1/3^ − d_P_) ~ 10 nm. As it was comparable to a transverse diameter of channel bundles, the formation of hybrid channels seemed probable as a result of the association of diamonds carrying ionic groups that were positively charged with a copolymer bearing negatively charged groups. This was consistent with the enhancement of scattering from the linear channel fragments with the diamond concentration (parameter A_C_) (Figure 10d), which indicated the growth of the total volume fraction of channels in the membranes. 

When the surface of the DND Z+ diamond framework became conductive, the membrane was transformed into a hybrid form; the polymeric ion channel bundles between the diamond chains also created a conductive interface with the copolymer. The results were achieved through the use of DND Z+ particles with a positive potential as modifiers of perfluorinated copolymers. Conversely, when we modified the same copolymer with negatively charged DND Z− diamonds carrying carboxyl groups, it resulted in a different structure of the composite, where the diamonds contributed minimally to the increase in conductivity of membranes (Figure 3c,d).

#### 3.2.2. Membranes with DND Z− Diamonds 

**Figure 14:**
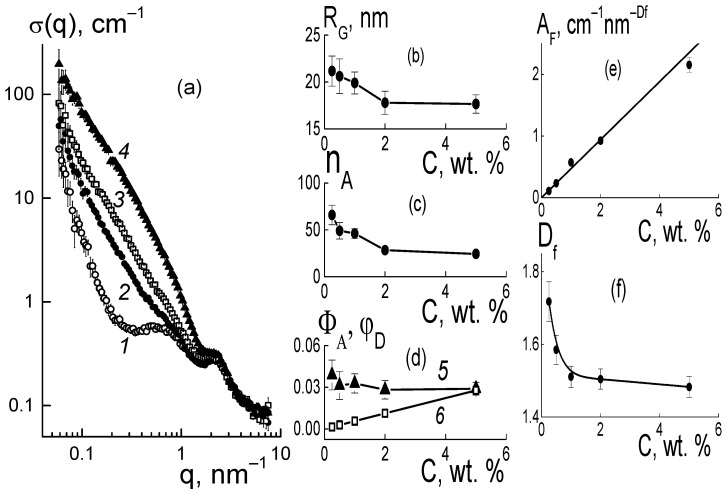
Scattering cross sections σ(q) for the pristine membrane (1) and composites with 0.25, 1.0, and 5.0 wt. % of DND Z− (2–4) vs. momentum transfer (q) (**a**). Concentration dependences of gyration radii R_G_(C) (**b**), aggregation numbers n_A_(C) (**c**), volume fractions Φ_A_(C), and φ_D_(C) of DND Z− in aggregates and over the sample (5,6) (**d**). Parameters of approximation for different cross sections Δσ(q,C) by fractal functions vs. DND Z− content: (**e**) parameter A_F_(C) and (**f**) fractal dimension D_f_(C) of diamond structures.

The SANS data for dry composites with DND Z− (Figure 14a) were similar to those for the membranes with DND Z+ (Figure 6a). In both cases, the initial copolymers and composites were prepared in similar ways, although the cross-sectional profiles of the initial samples in the low-momentum range (q ≤ 0.1 nm^−1^) were slightly different (Figure 6a and Figure 14a); this is related to spatial scales of tens or more nanometers and was not as significant in our work. We focused on the nature and mechanisms of channel formation and ordering in the composites at scales of ~10^0^–10^1^ nm, which has not been studied much to date.

According to the data in Figure 14a, the cross sections of composites with DND Z− at momenta q ≤ 0.1 nm^−1^ were approximately two times less than those of the samples with DNDZ+ (Figure 6a). This could be attributed to the mutual repulsion of the copolymeric and diamond ionic groups carrying negative charges. Such interactions are less conducive to extended chain assembly, but stimulate the segregation of DND Z− particles into submicron(micron)-sized globules detected on the surface of composite films by atomic force microscopy (AFM) [31]. In our neutron studies, such large aggregates were beyond experimental resolution, which was limited by the observation of structures with scales ~2π/q ~ 10^0^–10^2^ nm.

Similar to what has been shown above, when analyzing diamond structures in composites, the contribution of the polymer matrix was subtracted from the cross sections, Δσ(q,C) = σ(q,C) − σ(q,C=0). At small momenta q < 0.1 nm^−1^, we used the Guinier approximation to treat the different data and to determine the gyration radii R_G_ and aggregation numbers n_A_ of the diamond aggregates (Figure 14b–d). These structures had gyration radii R_G_ ~ 20 nm (Figure 14b) comparable to the sizes of the DND Z+ aggregates (Figure 7a).

A comparison of the data (Figure 7a,b and Figure 14b,c) for the composites with DND Z− and DND Z+ showed that in the samples with DND Z− particles, both the size and mass of aggregates (R_G_, n_A_) decreased more strongly than in samples with DND Z+. In the DND Z− aggregates, the volume content of diamonds, Φ_A_~3–4%, dropped to the level of the total fraction of diamonds φ_D_ in the sample with 5 wt. % modifier (Figure 14c). This fact was associated with the low fractal dimension of chains in the aggregates found from the approximation data at q~0.1–0.4 nm^−1^ using the function Δσ(q,C) = A_F_q^−Df^ with parameters A_F_(C), D_f_(C) (Figure 14e,f).

Similar to the samples with DND Z+ (Figure 7a), the parameter A_F_(C) = α_F_·C was found to be proportional to the modifier content (Figure 14f). However, factor α_F_ = 0.47 ± 0.02 cm^−1^ nm^−Df^ was twice as high as α_F_ = 0.24 ± 0.01 cm^−1^ nm^−Df^ in the membranes with DND Z+. A difference resulted from a lowering of the fractal dimension of DND Z− chains. This affected the coefficient α_F_ ~ 1/r_C_^Df^, which depended on the correlation radius of particles (r_C_). Thus, DND Z− diamonds formed chains with smaller indices D_f_(C) ~1.5–1.7 (Figure 14f), comparative to D_f_(C) ~ 2.2–2.4 in the DND Z+ structures (Figure 8b). 

In the composites, DND Z− particles formed linear, but not branched, chains with an excluded volume conformation, with a fractal exponent of ~ 5/3 [51]; these chains created nano-sized rarefied aggregates that coexisted together with dense submicron(micron) globular formations that were detected earlier by AFM at the surfaces of the membranes with DND Z− (1; 2 wt. %) [31]. Altogether, the neutron and AFM data [31] showed the whole structural organization of diamonds in the copolymer matrix in a wide range of spatial scales ~10^0–^10^3^ nm.

**Figure 15:**
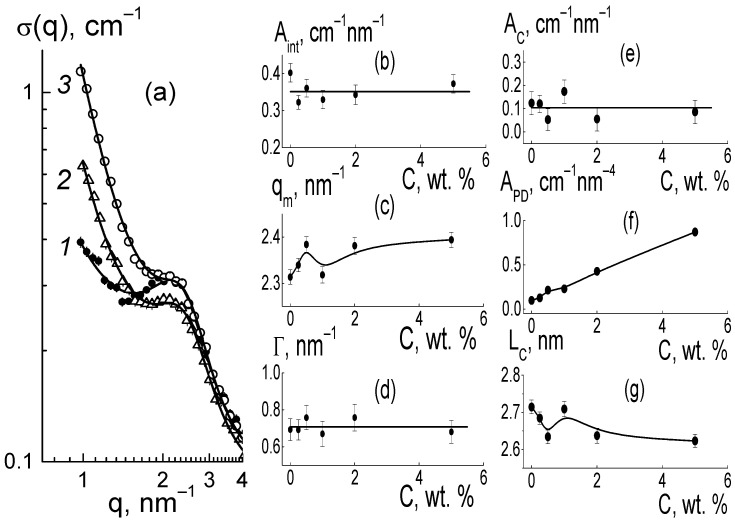
Approximation of cross sections for the pristine membrane (1) and composites with 2.0 and 5.0 wt. % of DND Z− (2,3) by Equation (3) (**a**). Structural parameters of the samples vs. diamond fraction (C): (**b**–**d**) amplitude A_int_(C), maximum position q_m_(C) and width Γ(C) of ionomer peak; (**e**,**f**) parameters A_C_(C), A_PD_(C) characterizing scattering from channel linear fragments and diamond-copolymer border; (**g**) channel packing period L_C_(C). Curves (**b**–**e**,**g**) denote data approximation using Function (4). Linear fit for A_PD_(C) data is shown (**f**). Lines (**b**,**d**,**e**) indicate the average levels for the parameters.

The bimodal character of the segregation of DND Z− diamonds limited their influence on the structuring of the copolymer, as evidenced by the behavior of the scattering cross sections σ(q,C) around the ionomer peak. Its profile obeyed Function (3) (Figure 15a), but the related structural parameters depended less on the diamond content (Figure 15b–g) than that in the samples with DND Z+ (Figure 10). 

In the samples with different fractions of DND Z− diamonds, the ionomer peak retained its amplitude A_int_(C) (Figure 15b) near the average level A_INTA_ = 0.35 ± 0.01 cm^−1^ nm^−1^. This reflected the weak influence of DND Z− particles on the polymer channel packing compared with the strong effect of the DND Z+ modifier. Meanwhile, the ionomer peak changed position q_m_ ~ 2.3–2.4 nm^−1^ (Figure 15c). The curve q_m_(C) demonstrated a minimum at point C = 1 wt. % (Figure 15c), which corresponded a transition to more straightened diamond chains with a lower fractal dimension, D_f_(C) ~ 1.7 → D_f_(C) ~ 1.5 (Figure 14f). Such changes only slightly disturbed channel packing. This can be seen from the stable peak width Γ(C) near the average level Γ_A_ = 0.71 ± 0.04 nm^−1^ (Figure 15d).

Parameters A_int_(C) and A_C_(C), characterizing the interference in scattering from the channels in bundles and the scattering intensity from the linear fragments of channels, also did not show significant variations (Figure 15b,e). It indicated the conservation of channel coordination in the bundles, with only weak changes in their packing period, L_C_ = 2π/q_m_ ~ 2.6–2.7 nm (Figure 15g), when a number of channels ν_C_ in a bundle and the transverse size of bundle L_B_ ≈ √ν_C_·L_C_ were quite stable. Then, we used the average magnitudes of the parameters, A_INTA_ = 0.35 ± 0.01 cm^−1^ nm^−1^, A_CA_ = 0.10 ± 0.02 cm^−1^ nm^−1^ (Figure 15b,e) to find the number of channels ν_C_ = (A_INTA_/A_CA_) + 1 = 4.4 ± 0.2, and the transverse size of a bundle L_B_ ≈ √ν_C_·L_C_ ≈ 6 nm.

We found that both hydrophilic diamonds (DND Z+ and DND Z−), forming branched or linear chains in the copolymer matrix, did not change the character of the copolymer chain assembly, where ion channels were packed into bundles consisting of the same number of coordinated channels, ν_C_ ~ 4–5, like in the pristine copolymer. The developed diamond-copolymer interface had an area proportional to the content of diamonds according to a variation in the A_PD_ parameter (Figure 10e and Figure 15f). Comparative with DND Z+ diamonds, the particles of DND Z− were less able to form additional ion channels with copolymer participation. This led to a significant difference in the conductivity of the compositional membranes with DND Z+ and DND Z− (Figure 2 and Figure 3).

It should be noted that, until now, there have been no attempts to compare the action of the detonation diamonds with different signs of the surface potential on the structure and conductivity of Aquivion^®^-type membranes. Here, we can mention only the article [52], where the authors detected substantial growth in the conductivity of Nafion^®^- and Aquivion^®^-type materials as a result of embedding of diamonds (0.26–4.0 wt. %) like our DND Z− particles. However, such an effect was observed only at a low humidity of composites (RH~10%), while it disappeared completely at higher water contents. These authors did not present any structural data to explain the conductivity changes in the composites. 

For a complete understanding of the mechanisms influencing the different types of diamonds on the membrane conductivity, the results of the composites with hydrophilic fillers (DND Z+ and DND Z−) were compared with the data for samples with hydrophobic fluorinated DND-F diamonds.

#### 3.2.3. Membrane Modified with Fluorinated Diamonds

**Figure 16:**
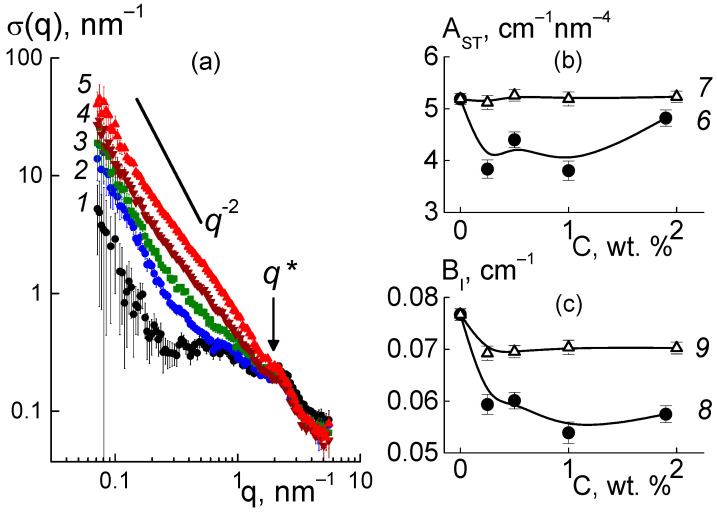
Neutron scattering cross sections σ(q) in the Aquivion^®^-type pristine membrane (1) and compositional membranes with DND-F diamonds (0.25; 0.5; 1.0; 1.9 wt. %) (2–5) vs. momentum transfer q (**a**). The behavior of the cross section ~q^−2^ for a Gaussian diamond chain is shown. The ionomer peak is noted at position q*~2 nm^−1^. The parameters A_ST_(C), B_I_(C) (**b**,**c**) are plotted vs. the content of DND-F (6, 8) and DND Z+ (7, 9) in the membranes.

As we have shown, fluorinated diamonds, even in small amounts (≤1 wt. %), blocked the conductivity of the Aquivion^®^-type membranes (Figure 2c) due to the interaction with the non-polar polymer backbone, perturbating their packing and disrupting the ion channels. We studied these structural changes using SANS on dry composites with different fractions of DND-F particles (0.25–1.9 wt. %) (Figure 16a). In the composites, the scattering cross sections at momenta q ≤ 0.3 nm^−1^ followed the ~q^−2^ law, as stated above for the samples with DND Z+ and DND Z−. Such a regularity in cross section behavior indicated a binding of DND-F particles into the chain structures. At the same time, there were packs of channels in the membranes, which were detected using the the ionomer peak at the position q* ~ 2 nm^−1^ (Figure 16a).

At high momenta q ≥ 3 nm^−1^, the effect of DND-F diamonds on ion channels was realized in the behavior of the characteristics A_ST_(C), B_I_(C) found from the approximation of q^4^σ(q) data by Function (7), and was compared with the characteristics of samples with DND Z+ (Figure 16b). Parameter A_ST_ = 2π[(ΔK_P_^2^)S_TP_+(ΔK_DP_^2^)S_TD_] included the contributions proportional to the areas S_TP_ and S_TD_ of the surface of polymer channels and diamond-copolymer interface. A small number of DND-F particles (C = 0.25 wt. %) caused a sharp decrease (~20%) in A_ST_(C) value and the channel area S_TP_ in the matrix. Further (C = 0.5; 1.0 wt. %), the A_ST_ values did not increase.

Consequently, the formation of a hydrophobic interface between the diamond particles and the copolymer occurred at the expense of reducing the network of channels. Partial reduction of A_ST_ at C = 1.9 wt. % can be attributed to the increased segregation of diamonds, the effect of which resulted in a weaker structure of the copolymer (Figure 16b).

It should be clarified that the formation of an interface between the diamonds and hydrophobic chains located on the diamond facets created some steric restrictions for the ionic groups in the chains. This did not allow them to form channels, although the segregation of groups into multiplets was possible [53,54]. As a result, a network of channels was fragmented, which led to a reduction in the adsorption capacity and, consequently, to a reduction in the conductivity of the composites (Figure 2c).

**Figure 17:**
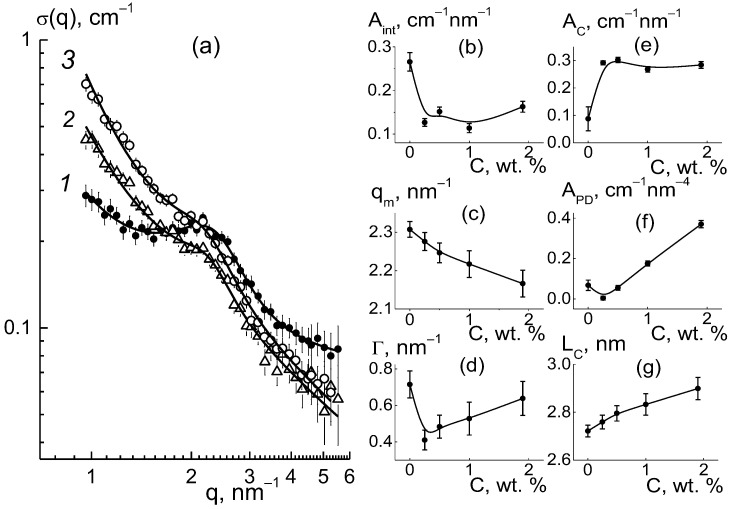
Scattering cross sections σ(q) vs. momentum transfer q around the ionomer peak area for the membranes with different DND-F fractions (C = 0; 1.0; 1.9 wt. %) (1–3) (**a**). The data are approximated using Function (3) (**a**). Characteristics of the ionomer peak vs. the content (C) of DND-F diamonds in the membranes: (**b**) amplitude A_int_(C); (**c**,**d**) position q_m_(C) and width Γ(C) of the peak; (**e**,**f**) parameters A_C_(C), A_PD_(C) characterizing scattering from linear fragments of channels and diamond-copolymer interface; (**g**) period L_C_(C) in the arrangement of channels.

This conclusion was confirmed by the cross-section B_I_(C) decrease (Figure 16c), which was directly related to a lack of bound water in the membranes. Subsequent analysis of the ionomer peak profile revealed specific structural changes in the membranes caused by the DND-F modifier (Figure 17).

DND-F diamonds introduced into the copolymer provoked a smoothing of the peak (Figure 17a) that reflected a weakening of short-range order in channel packing. The changes in the parameters of the ionomer peak profile approximated by Function (3) (Figure 17b–d) confirmed this assumption. A small number of diamonds in the copolymer (0.25 wt. %) caused a sharp decrease in the interference of scattering on ion channels when the amplitude A_int_ of the peak decreased twice, which then changed a little with the increase in diamond fraction (Figure 17b). At the same time, the total scattering from linear objects, such as ion channels and possible extended stacking defects of fluorocarbon chains, characterized by the parameter A_C_(C), sharply increased at the minimum content of diamonds (C = 0.25 wt. %) and remained quite constant at higher amounts, C = 0.5–1.9 wt. % (Figure 17e). 

Such opposite changes in parameters A_int_(C) and A_C_(C), upon the modification of membranes with fluorinated diamonds, were similar to what was observed in composites with DND Z+ particles (Figure 10a,d) when hydrophilic diamonds formed hybrid conducting channels together with copolymer ionic groups. In the case of DND-F, the embedded particles became incorporated into fluorocarbon chain packs. Hence, the action of particles on the matrix structure was extended at least until a scale of polymeric domains ~10 nm in size.

With an already low content of diamonds (0.25 wt. %), their numerical concentration ~0.3 × 10^17^ cm^−3^ determined a short average spacing between the diamond particles (~30 nm) that was quite comparable to the scale of copolymer domains. Hence, their arrangement was influenced by the modifier.

Indeed, DND-F particles with a size ~4.5 nm, an order of magnitude larger than the transverse chain packing period, caused the formation of extended linear stacking defects of chains that were revealed in the growth of the cross section (~1/q) with the A_C_(C) coefficient (Figure 17e). Also, the interface area became larger, proportional to the concentration of diamonds (A_PD_ parameter) (Figure 17f). This prevented the ordering of polymeric ion groups into the channels, which resulted in a sharp weakening in the interference peak A_int_(C) (Figure 17a).

**Figure 18:**
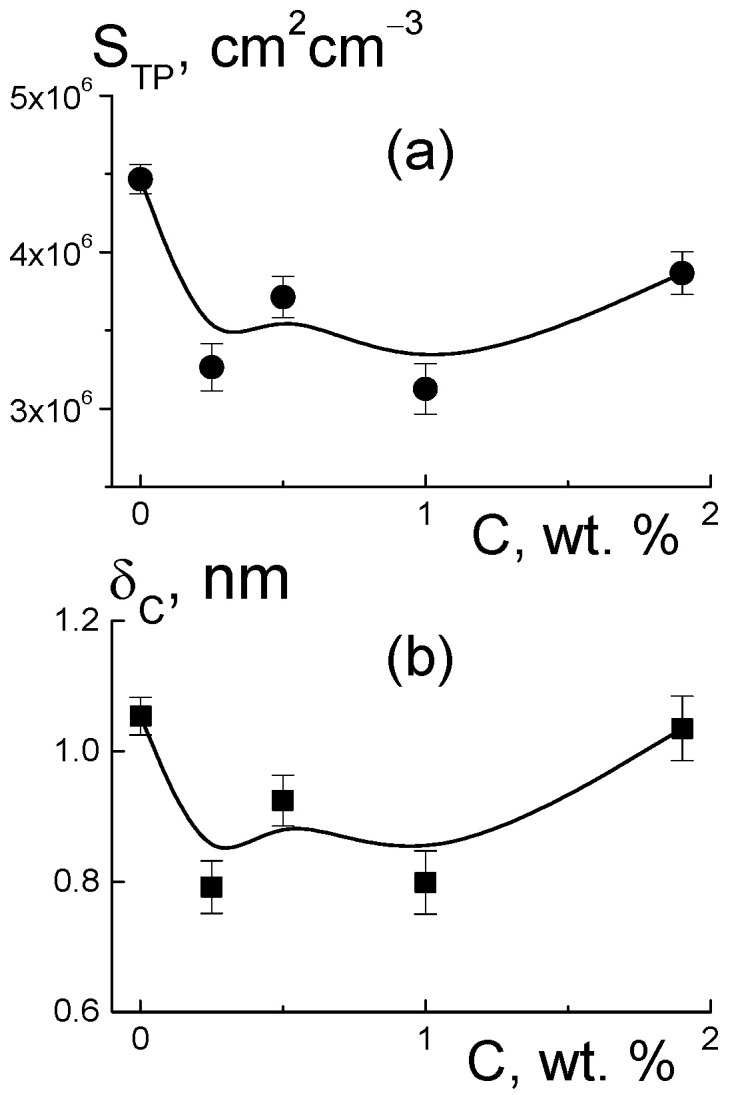
Total area S_TP_(C) and channel diameter δ_C_(C) vs. DND-F diamond fraction in membranes (**a**,**b**).

When the membranes were enriched with diamonds, the peak shifted to a smaller momentum, first narrowing, but then expanding (Figure 17c,d). This indicated a loosening of dense packing in nonpolar chains around the ion channels when the period in channel arrangement was grown (L_C_ ~ 2.7 nm → L_C_ ~ 2.9 nm) (Figure 17g). Such a variation in the period was opposite to one observed in the composites with DND Z+ (Figure 10f). Moreover, a small amount of DND-F (0.2 wt. %) provoked a reduction in channel surface area S_TP_ and diameter δ_C_ = S_TP_L_C_^2^/π by ~20% (Figure 18).

Compared with DND Z+ particles (Figure 13b), fluorinated diamonds (C ≤ 1 wt. %) induced a two-fold stronger effect on channel squeezing. But, with a higher content of DND-F (1.9% wt.), channel expansion with a partial restoration of the total surface area was detected (Figure 18). This phenomenon seems to be associated with the segregation of some diamonds in the matrix, when their influence on the copolymer weakened.

The analysis of the structural effects in hydrophilic (DND Z+ and DND Z−) and hydrophobic diamonds (DND-F) revealed different mechanisms of action on the copolymer matrix. It has been established that DND Z+ diamonds are the most effective for proton conductivity improvement in membranes. As a result of attraction to the ionic groups of the copolymer, DND Z+ particles created additional ionic channels, which contributed to the conductivity of the membranes. DND-F diamonds, with a hydrophobic surface, interacted predominantly with non-polar copolymer chains, creating a non-conductive interface along with some isolated ion multiplets, instead of channels, which destroyed the conductive channel network.

All of the preceding analyses on the electrophysical and structural features of membranes with different fillers allowed us to generalize existing conceptions concerning some basic principles regarding the formation of ionic channels in proton-conducting perfluorinated copolymers. 

#### 3.2.4. Micellar Models for Membranes

The results obtained provide grounds to consider cylindrical inverted micelles with a shell of hydrophobic chain fragments around the ion channel as the key element of the membrane structure. As a result of micelle packing, a matrix with a system of channels was created, the polar surface of which formed the boundaries of water inclusions in the surrounding hydrophobic polymer matrix.

**Figure 19:**
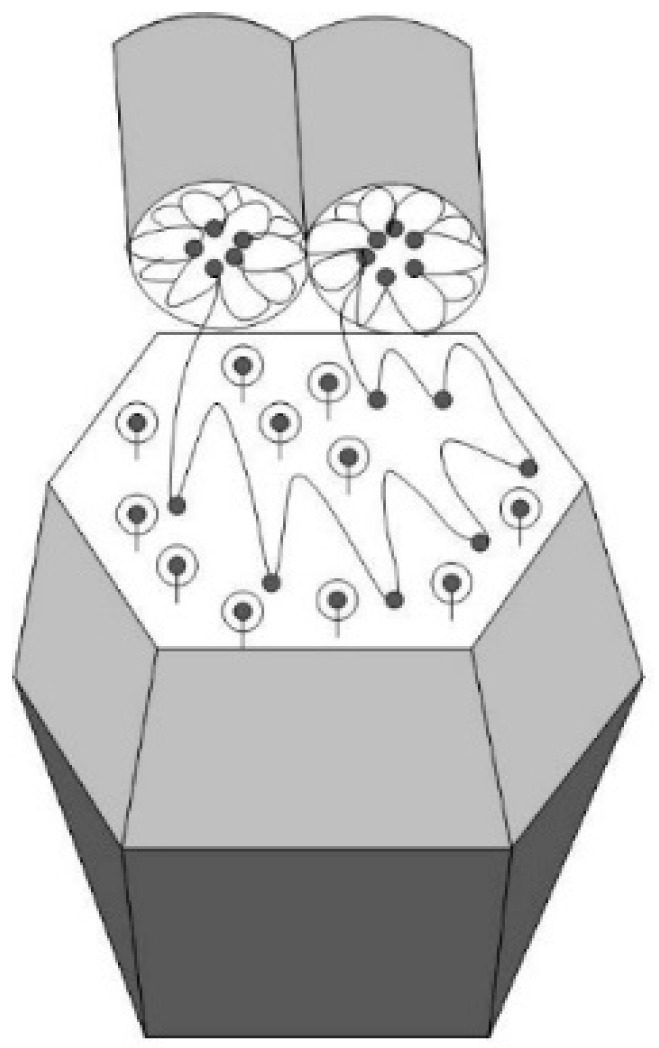
Adsorption of a copolymer chain in the conformation of cylindric micelle on a diamond face and the binding of ion channels when lamellar chain fragments are attached to the diamond surface. Black dots in folded copolymer chains denote ionic groups. Own ionic groups of the crystal surface are shown (circles with dots).

In the composites with hydrophilic diamonds (DND Z+ and DND Z−), we considered such micelles as being in contact with crystal facets when the fragments of macromolecules achieved both micellar and lamellar conformation, and the facets served to connect ionic channels (Figure 19).

As it is known, in solutions with low molecular weight surfactants, the size and geometry of micelles are determined by the length of the surfactant molecules and their concentration [55,56,57]. Similar to this, a membrane-forming copolymer can be considered as a polymerized surfactant composed of a sequence of fragments. Each fragment includes a short hydrophobic chain with a hydrophilic unit carrying a side chain with a terminal ionic group. This specifies the equivalent weight parameter (EW), e.g., for Aquivion^®^, it is usually EW ~ 500–1000 g-eq/mol. Such a molecular structure has all of the necessary preconditions for the conformation of inverted cylindric micelle in a nonpolar surrounding, which is, in our case, a bulk copolymer matrix.

We used a copolymer of Aquivion^®^-type with an equivalent weight EW = 890 g-eq/mol. This chain fragment included a number of fluorocarbon units (on average ≈ 6.12) and one unit with the ionic group. The total number of units n_U_ ≈ 7.12 corresponded to a chain with a contour length L_m_ = n_U_l_U_ ≈ 1.8 nm composed of units with a size l_U_ = 0.25 nm, similar to that generally found in carbon chain polymers.

Here, we assumed that such chains were folded when forming cylindric micelle (Figure 19) with a diameter L_C_ ≈ 2.7 nm, according to neutron scattering data for the channel packing period in the membranes (Figure 10f). Then, the difference δ_C_ = L_C_ − L_m_ ≈ 0.9 nm provided the estimate of the diameter of channels covered with ionic groups with a size D_IG_ ≈ 0.3 nm. This implieds the number of groups along the perimeter in the cross section of the channel, n_TR_ = π(δ_C_ − D_IG_)/D_IG_ ≈ 6.

To achieve the minimum energy for the dipole interactions, the electric dipoles of the groups were oriented “head-to-tail” and formed a spiral “winding” in the form of a sequence of dipoles and a helix pitch of about twice the chain diameter (~1 nm).

This corresponded to hollow polymerized micelle, formed by joining initially flat multiplets (~6 groups) into a cylindrical entity. Here, an analogy can be drawn between such micelles and structures synthesized from low-molecular-weight surfactants that form cylindrical micelles in solutions, which are stabilized by chemically cross-linking surfactant molecules [58,59,60].

This also resembled a coil-globule transition observed for sulfonated polystyrene in chloroform as result of the electric dipole interaction of SO_3_Na ionogenic groups in chains (1.35 mol. %). Increasing the number of ionic groups to 2.6 mol. % led to packing the ionomer chain into a vesicular structure with a solvent inside (vesicle) [61].

The ordering process for perfluorinated copolymers at the initial stage can be represented as a result of the segregation of several neighboring ionic groups interacting through electric dipole moments and hydrogen bonds, accompanied by the docking of their hydrophobic tails, radially diverging from the multiplet. A cylindrical polymer layer with a thickness of L_m_/2 and a width corresponding to double the diameter of the chain was created around it. Adjacent sections of the chain were attached to such a structure according to the same principle of segregation of ionic groups and sequential stacking of hydrophobic chain fragments.

As a result, the copolymer turned into a cylindrical micelle with a cavity covered with ionic groups and a polymer shell with a local orientational order of chain fragments. The relationship between the inner and outer diameters of the micelle was determined using the equivalent weight of the copolymer. Because EW was fixed, the micellar structure remained stable under various conditions (interaction with solvents and modifier particles, mechanical deformations, etc.), while the ion channels underwent an expansion by membranes hydration [62]. As shown in [63,64], for Aquivion^®^-type membranes (EW = 790; 870 g-eq/mol) without nanoparticle fillers, through uniaxial stretching of the membranes, their structure was rearranged, but the position of the ionomer peak did not change, which indicated high stability of the channel structures.

As for the structural changes, the following question arises: how can diamond particles (~4.5 nm) significantly larger than the diameter of the ion channels be incorporated into the copolymer matrix? Obviously, a hydrophilic surface of DND Z+ or DND Z− crystals with grafted ionic groups (H, OH or COOH) is capable of adsorbing polymeric acid groups SO_3_H due to dipole interactions and hydrogen bonds. Hence, the chain fragments with the associated SO_3_H groups should be stacked perpendicular to the diamond surface and form a flat lamella, but outside the crystal facet, the chain takes the conformation of a cylindrical micelle. If the fragments of several chains condense on a diamond facet, a planar conduction channel linking ion channels can be created using different chains.

**Figure 20:**
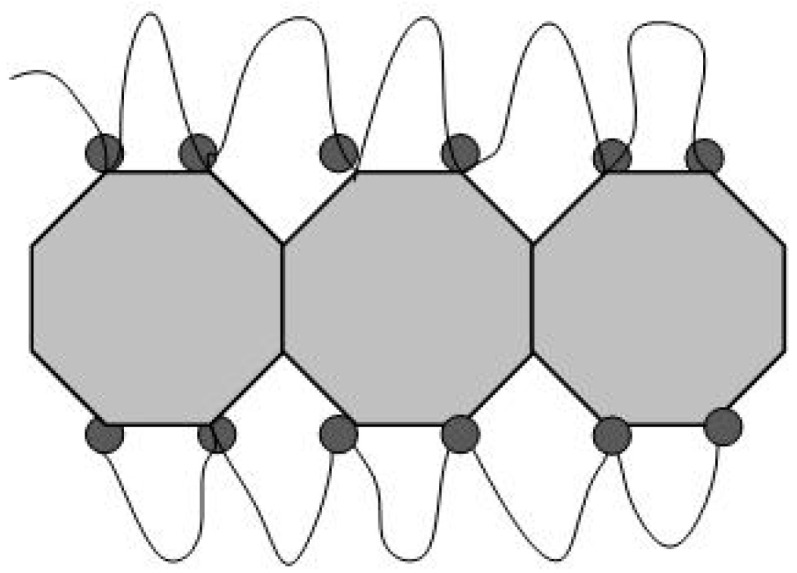
A cross-section of a micelle fragment with a diamond core and a polymer shell.

A positive effect of polymer modification with diamonds was expressed not only in the appearance of additional sites for protons due to ionic groups on diamonds, but also in the formation of links for ion channels (Figure 19). It is worth paying attention to the fact that the diamonds were organized in the chains that adsorbed SO_3_H groups, which made an adjacent lamellar layer from the polymer. In this way, hybrid micelles formed on the diamond core (diameter ~ d_P_ ~ 5 nm) (Figure 20) when conducting diamond-polymer interface and lamellar polymer shell had the same thickness, Δ_DP_ ~ L_m_ ~ 1 nm, and the outer diameter of such micelle was twice the size of the diamond, D_DM_ = (d_P_ + Δ_DP_ + L_m_) ~ 9 nm.

Packing the micelles in a matrix suggested their local orientational order with a gap between the diamond surfaces 2(Δ_DP_ + L_m_) of ~ 4 nm. This length determined the upper limit for the concentration of diamond particles in membranes, N_P_* ~ 1/(d_P_D_DM_^2^) ~ 2.7 × 10^18^ cm^−3^, when the entire polymer was completely consumed to coat the diamonds with a mass fraction of ~23% (volume content ~13%).

Although both hydrophilic fillers (DND Z+ and DND Z−) had a trend to form conducting interfaces adjacent to the diamond chains, this is actually preferable for positively charged DND Z+ particles.

**Figure 21:**
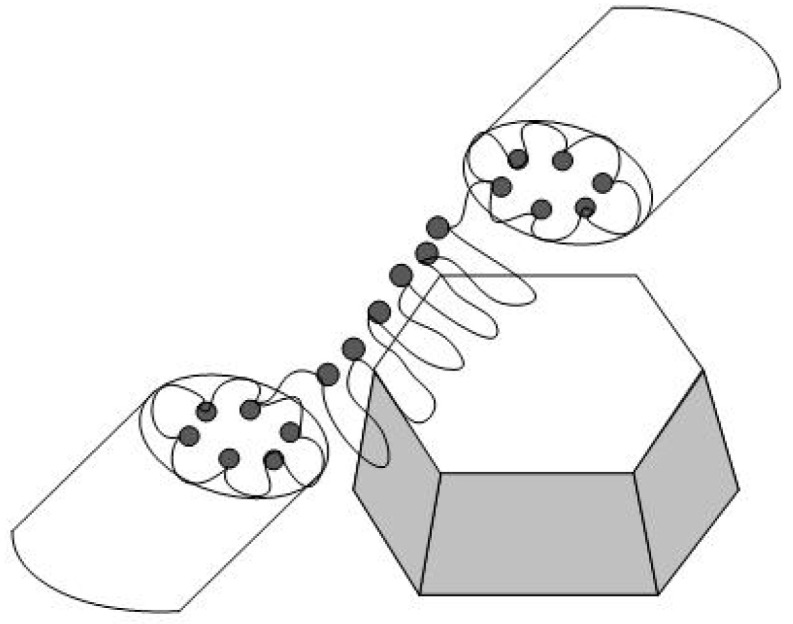
Interruption of the ion channel upon partial adsorption of a chain on a diamond face.

In contrast with this case, predominantly hydrophobic DND-F particles did not bond to polymeric ion channels, but rather interrupted them because of the adsorption of backbone chains on crystal facets, when their ionic groups could not participate in conducting an interface (Figure 21).

We should note, because of the very long total channel length per membrane’s unit volume L_ct_ ≈ 2.3 × 10^13^ cm/cm^3^, fluorinated diamonds (1 wt. %) could reduce it only slightly, ΔL_ct_/L_ct_ ≈ 0.24%. Here, we calculated L_ct_ assuming that every six ionic groups formed a multiplet, which provided a contribution of ~2d_pol_ ~1 nm to the channel length, and the fluorinated diamonds destroyed small channel fragments with a particle diameter size of d_P_ ≈ 4.5 nm. Therefore, the diamond’s effect on membrane conductivity could be a consequence of channel disconnection, but not shortening.

**Figure 22:**
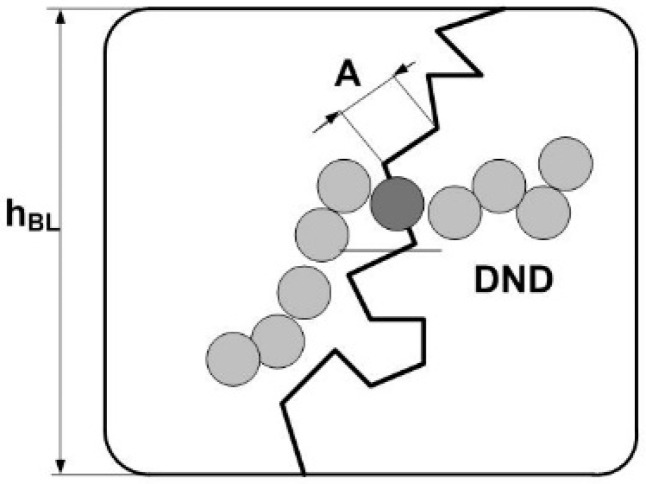
Fragmentation of micellar channel upon interaction with fluorinated diamond.

To assess the probability of channel fragmentation in the membrane, we compared the number (n_BL_) of channels penetrating a polymer block (size h_BL_ ~ 150 nm) in the membrane and the number of diamond particles (concentration N_DND_) in a block, N_DBL_ = N_DND_h_BL_^3^ (Figure 22).

A channel in the form of a Gaussian chain composed of n_A_ segments (A ~ 10 nm in size) had a contour length L_1_ = A·n_A_ and squared chain diameter h_BL_^2^ = n_A_·A^2^. With the total length of channels in the block L_tBL_ = n_BL_·L_1_ = h_BL_^3^·L_ct_, the number of channels, n_BL_ = h_BL_·L_ct_·A ≈ 340 does not exceed the number of diamond particles, N_DBL_ ≈ 400, in each polymer block at the filler fraction C = 1 wt. %. Thus, most channels should be destroyed by diamonds, and membrane conductivity is expected to be decreased in proportion to exp(-N_DBL_/n_BL_) ≈ 0.30, which is a probability of a particle not entering a channel. This estimate agreed with a low conductivity level for such a modified membrane, ~26% of the conductivity of the pristine membrane.

Our studies of Aquivion^®^-type composites concerned the problems regarding membrane modification with diamonds carrying hydrogen or fluorine atoms, hydroxyl, or carboxyl groups at the surface. However, there are other methods of improving membrane functional properties, for example, using diamonds with sulfonic acid groups similar to those in copolymer side chains.

In a recent work [11], an Aquivion^®^-type material was filled with diamonds with grafted sulfonic acid groups (0.5–2.0 wt. %) to stimulate proton conductivity, while maintaining good mechanical properties for the membranes; furthermore, their structure was not noticeably disturbed by such a modifier. Meanwhile, DND Z+ diamonds provide an increase in conductivity through heating the from 20 to 50 °C (Figure 3), approximately twice as much as that observed under the same conditions for membranes with sulfonated diamonds [11].

In connection with the obtained results, we have to stress the general problems of comprehension for the structuring mechanism of membranes from perfluorinated copolymers with nonpolar fluorocarbon backbones and side ionic groups. Attempts have been made to understand the patterns of formation of membrane morphology based on studies of the behaviors of perfluorinated copolymers in solutions [65,66,67,68].

In polar solvents (water-alcohol mixtures), the structuring of perfluorinated copolymers (Nafion^®^) occurs by combining fluorocarbon backbones into fibrils [65,66,67,68]. Neutron experiments [68] have established that in solutions and gels, Nafion^®^- and Aquivion^®^-type macromolecules form highly elongated fibrils with hexagonal ordering transversely to their axis. Fibrils with a perfluorinated core and charged ionic groups on the surface had a length of ~1000 nm and a diameter of 4–6 nm when the transverse size of the aggregates depended more on the surface tension than on the dielectric constant of the solvent.

The fibrillar organization of copolymers in solutions, as in the formation of cylindrical surfactant micelles [69], does not provide compelling reasons to expect that fibrils with a hydrophobic core and ionic groups on the surface will predetermine the morphology of a dry membrane, implying only the transformation of original fibrils into an inverted form during the deposition of the copolymer from the solution onto solid substrates, the removal of the solvent, and the subsequent heat treatment in order to achieve an equilibrium structure.

To create and stabilize a network of ion channels, the membranes were heated above the glass transition region and were annealed (T ~ 120 °C > T_g_ ~ 100 °C for Nafion^®^) [70]. The achieved equilibrium structure of Nafion^®^ membranes was dominated by cylindrical (curved) fibrils with a diameter of ~3 nm [70]. During annealing, the fibrils were oriented and straightened in an electric field to increase the proton conductivity of the material. Transmission electron microscopy data [70] did not show obvious accumulations of ionic groups with a high contrast (lead modification) on the surface of the fibrils. This indicated a structural transformation of the copolymer during deposition from the solution, formation, and annealing of the membrane film.

In fact, heat treatment provided high segmental mobility to the copolymer chains, facilitating the transition of macromolecules to an energetically favorable (equilibrium) conformation, as determined, first of all, by dipole interactions of the ionic groups neighboring in the chain with the formation of multiplets with the participation of hydrogen bonds [71]. In turn, sequential linear binding of multiplets transformed macromolecules into cylindrical fibrils with a central ion channel (~1 nm in diameter) and a non-polar shell (outer diameter ~3 nm) in the form of folded packing chain fragments between groups (Figure 19 and Figure 21). In this case, the size of the copolymer’s statistical segment did not exceed the length of the chain fragment between the ionic groups (~2 nm with an equivalent weight of ~1000 g-eq/mol), which were defects in the linear chain and contributed to its fracture. Thus, the persistent chain length of ~1 nm (half the segment length) determined the thickness of the polymer shell, with segments radially arranged from the central channel in the cross section of the micelle, and the outer diameter of the micelle being ~3 nm, which was the sum of the channel diameter and twice the shell thickness.

It should be noted that another (spherically symmetric) option for the formation of a micelle from a high-molecular copolymer would be geometrically impossible, as then the size of the central area (~10 nm) filled with ionic groups would be larger than the length of the chain fragment between the groups (~2 nm). Although the formation of an inverse micelle with a central cavity is possible according to Gierke [12], this model has not been confirmed experimentally [5,21,22,23,24,25,26,27,28,29,30].

The formation of monomolecular cylindrical micelles is favored by segmental diffusion inside polymer coils, when contacts of neighboring and more distant ionic groups occur, and their binding by dipole forces and hydrogen bonds leads to segregation of groups from non-polar fragments. As a result of primary self-organization at the molecular level and transformation into micelles, macromolecules create packings with short-range orientational orders (bundles).

In contrast with such a sequential formation of membrane morphology, the authors [27] proposed an alternative version of supramolecular ordering, when ion channels form in the gaps between parallel polymer chains, which serve as a stabilizing framework and are combined into bundles (fibrils). This model [27] is valid for rigid chains in a strained conformation, when the association of ionic groups due to dipole forces entails the assembly of chains along the perimeter of the channel. The formation of such structures in membranes obtained by the precipitation of a copolymer from a solution would mean the transformation of already existing fibrils (~1000 nm long, ~4–6 nm in diameter) [68] into inverse micelles as a result of the rotation of the chains around the fibril axis and the movement of ionic groups into the channel surrounded by fluorocarbon chains.

However, such large linear micelles bound into bundles have not been observed in membranes. In contrast, transmission electron microscopy (TEM) demonstrated curved micelles with a diameter of ~3 nm and a length no more than an order of magnitude greater than the diameter [70], consistent with the model of monomolecular cylindrical micelles (Figure 19 and Figure 21). This was also supported by atomic force microscopy data (measurements of topography and local current from the surface) [70]. The authors of [72] identified conducting structures on the surface of a moistened Aquivion^®^-type membrane at a scale of tens of nanometers as rolled up conductive lamellae with water conduction channels several nanometers thick enclosed between them, but the mechanism of the appearance of channels with shells of rolled up lamellae was not discussed.

The idea of folding lamellas was used in this work. We assumed that the macromolecule from the flat lamella conformation was twisted into a cylindrical helix with an ion channel along the axis (Figure 19 and Figure 21). Thus, a copolymer with an equivalent weight of ~1000 g-eq/mol and molecular weight ~10^6^ Da was converted into a micelle with a length of ~100 nm, when ~10 ionic groups were placed on the transverse perimeter of a channel with a diameter of ~1 nm. When micelles were packed in parallel with a period around their diameter of ~3 nm, bundles were formed; from this scattering, a corresponding ionomer peak was observed (Figure 9, Figure 15a and Figure 17a). The transverse and longitudinal dimensions of the micelles were in good agreement with the neutron and synchrotron scattering data, as well as transmission electron and atomic force microscopy [5].

Hypothetically, the assembly of a micelle with a central ion channel is possible as a result of the association of ~10 macromolecules in a flat lamella conformation, when the number of ionic groups on the transverse perimeter of the channel is also ~10. The length of the assembled micelle will reach ~10^3^ nm, and it will be a rigid rod. In this case, the polymer matrix will consist of micron-sized domains in the form of parallel-packed micelles. However, such large micelles and domains have not been observed in membranes [5]. Thus, the copolymer ordering model, based on the mechanism of conformational rearrangement of a flat macromolecular lamella into a cylindrical micelle, allowed us to combine the ideas of the lamellar model [73], which specifies the packing period of ion channels by the chain length between the ion groups and the channel size, and the model [27] of bundles of cylindrical nano-sized water channels with stabilizing polymer frames.

It is important to note that it is energetically favorable in a polymer matrix to form precisely cylindrical monomolecular micelles with ionic groups almost completely shielded from the weakly polar polymer medium. In this case, the assembly of groups into sequentially connected multiplets (channels) has no steric restrictions and provides a high density of group placement on the channel surface. At the same time, narrowing of the channels is achieved, when, with a channel diameter of ~ 1 nm, an increase in the diffusion and proton conductivity of water is observed by several orders of magnitude in comparison with bulk water [74]. The introduction of diamonds into the copolymer creates conditions for the formation of ionic conduction channels along the crystal facets, when adjacent fragments of the copolymer transform into a flat lamellar conformation (Figure 19, Figure 20 and Figure 21). Thus, the copolymer matrix combines different packaging options for the copolymer to form ion channels.

It is of interest to compare the results obtained for composite membranes based on perfluorinated copolymers with short chains with data for perfluorinated sulfonic acid membranes of the Nafion^®^-type with long side chains when these materials are modified with various nanoparticles in connection with the prospects for the development of hydrogen energy and the tasks of creating materials for the entire technological chain, including hydrogen production, purification, storage, and electricity generation [75]. Ion exchange membranes [75] based on perfluorosulfonic acids are characterized by a high conductivity and selectivity, especially when using copolymers with a short side chain. However, perfluorinated membranes are very expensive and can only operate effectively at high humidity and temperatures up to 100 °C; this introduces the problem of poisoning of catalysts with carbon monoxide, the sorption of which at temperatures up to 120 °C is virtually irreversible. Hence, there is a need to develop and modify membrane materials by introducing nanoparticles to improve proton conductivity and selectivity during proton transfer.

The authors of [76] studied the mechanism of the effect of functionalization of the surface of Nafion^®^-type membranes and their modifications containing SiO_2_ nanoparticles (3 wt. %), propyl, 3-aminopropyl, and 3,3,3-trifluoropropyl, on the physicochemical and electrochemical properties of membranes. It has been shown that filling Nafion membranes with negatively charged nanoparticles (3 wt. % SiO_2_; 5 mol. % 3,3,3-trifluoropropyl) led to an increase in their conductivity by 20% in all cases, except when 3-aminopropyl was used, which imparted nanoparticles with a positive charge, and the selectivity of membranes increased in all composites, which was explained by the transformation of the mesoporous structure of the membrane into a microporous one. The effect of nanoparticles on membrane conductivity was mainly caused by the additional (positive) spatial charge introduced into the pore solution and onto the membrane surface by the electrical double layer surrounding the nanoparticles. The greater the surface charge density of nanoparticles and the smaller their size, the stronger the effect. Therefore, the sample doped with SiO_2_ and 3,3,3-trifluoropropyl showed the highest conductivity and current density at a low fixed voltage.

The possibilities for improving the hydrophilic and conductive properties of perfluorinated membranes, in particular Nafion^®^ 117 and Nafion^®^ 115 materials in comparison with CMX (Neosepta) and Fuji-CEM 80050 (Fujifilm) membranes used in RED (reverse electrodialysis) installations, were discussed in the literature [77]. The inclusion of inorganic nanoparticles (hydroxides of polyvalent elements of silicon, zirconium, etc.) into their structure led to a decrease in the gas permeability and methanol permeability of hybrid membranes for a variety of applications, including metal-ion batteries and redox cells. There have been known attempts to use nanoporous membranes with a high selectivity and permeability for the RED process through the use of silicon oxide nanotubes and anodic aluminum oxide.

The authors of [78] studied the effect of ultrasonic treatment for Nafion^®^ copolymer solutions in the presence of SiO_2_ nanoparticles regarding the characteristics of Nafion+SiO_2_ cast hybrid membranes. Ultrasonic treatment of polymer solutions reduced the length of the macromolecules and the number of sulfonic groups. When the polymer solutions were treated with ultrasound in the presence of SiO_2_, additional chain cross-linking occurred when SiO_2_ interacted with the sulfonic groups of the copolymer. As a result, up to 20% of –SO_3_H groups were excluded from the ion-exchange mechanism, and the temperature of the destabilization of ion clusters decreased; but, at the same time, the hydrophilic filler was included in the pores, the overall water absorption of the hybrid membranes increased, larger pores formed in them, and the connecting channels became wider, which facilitated the transport of protons and led to greater proton conductivity. Thus, ultrasonic treatment when dispersing filler nanoparticles in Nafion^®^ solutions made it possible to obtain hybrid membranes with improved transport characteristics.

In membrane technologies, the most important task remains to increase the chemical resistance of proton exchange membranes [79]. Membrane life limitations arise from free radicals in membrane processes. Nanoparticles can serve as free radical scavengers. The authors of [79] described a one-step method for preparing in situ hybrid membranes based on the copolymer Nafion^®^ 117 and sulfonic or phosphoric acid functionalized with cerium oxide. The conductivity of membranes containing sulfonic acid modified with cerium exceeded that of the original Nafion^®^ 117 membrane at a relative humidity RH = 30% [79]. In this regard, it is known that membranes initially treated with an oxidizing agent have a better conductivity with a lower permeability to water [80]. The effect of modifying Nafion 117 membranes with 3,4-ethylenedioxythiophene (EDOT) and hot pressing of hydrogen-oxygen membrane-electrode assemblies increased the efficiency of proton exchange membrane fuel cells [80].

Another way to improve the electrical transport characteristics of membranes was to plasticize the polymer electrolyte with high-boiling bipolar aprotic solvents, as shown by the example of the lithiated Nafion^®^ 115 membrane plasticized with sulfolane (SL), ethylene carbonate (EC), and diglyme (G2) [81]. A Nafion^®^ 115 membrane in lithiated form (Li-Nafion) was plasticized with a mixture of ethylene carbonate (EC) and sulfolane (SL) to obtain a polymer electrolyte with single-lithium conductivity and increased stability [82]. The electrochemical properties of swollen Li-Nafion with non-volatile binary plasticizer EC/SL were found to be suitable for practical applications in lithium batteries [82].

The authors of [83] introduced ferrocyanide-ferrocyanide Fc (II)–Fc (III) particles into proton exchange membranes (PEM), perfluorosulfonic acid (PFSA), and sulfonated hydrocarbon membranes. The particles participated in the redox cycle and scavenged radicals. Composite membranes based on Nafion^®^ and Aquivion^®^, as well as hydrocarbon SPEEK, SPSf, SPS, and SPN, had increased chemical stability and durability for operation in fuel cells compared with the original membranes and were superior to the composite membranes with cerium Ce^3+^ ions traditionally used as antioxidants. The proposed strategy is considered to be a universal one for improving the chemical oxidation resistance of hydrocarbon-based PFSA and PEM membranes.

In connection with the operation of fuel cells, attention has been paid to the problem of hydrogen crossover, which affects the durability of hydrogen fuel cells [84]. The authors [84] discussed the effect of hydrogen crossover on the components and characteristics of fuel cells; analyzed the factors of structural permeability and the reasons for the destruction of the membrane, as well as ways of increasing its durability, including through chemical cross-linking, but this led to a loss of conductivity. It was also proposed to add stable porous materials to the membrane to reinforce it. To improve the chemical stability and durability of the membrane and reduce hydrogen crossover, methods were considered in order to change the chemical structure of the membrane and reduce the quantity of free radicals by introducing scavengers (inhibitors), such as metal oxides and their complexes (CeO_2_, ZrO_2_, MnO_2_, etc.).

Our analysis of works in the field of ion-exchange membranes [75,76,77,78,79,80,81,82,83,84] showed high research activity and achievements in the field of improving the functional properties of membranes in various ways, including using nano-sized modifiers, but without the use of nanodiamonds, despite the beneficial properties of such crystalline particles. These particles are chemically inert and heat-resistant, and are capable of carrying grafted ionic groups that create a charge on the surface of the particles, the sign and magnitude of which can be set during the modification process. Hence, the novelty and significance of the results obtained in our work on the modification of diamonds and the preparation of composite membranes with different concentrations of diamonds is obvious.

The membrane modification methods developed in our work can be compared, in particular, with the introduction of nanofibers into proton exchange membranes (PEM) [85], where the fibers are able to form a stabilizing framework and long-range channels for proton transport, while reducing fuel crossover. This kind of structural design using functionalized fibers that create interconnected channels (networks) for proton transfer is attractive for membrane technologies, but requires significant improvements in the methods for obtaining fibers with desired structural and physicochemical characteristics. A related area can be considered as the development of methods for modifying membranes with polymers. The authors of [86] synthesized sulfonated poly(indene) (SPInd, degree of sulfonation 35%; 45%) and mixed it with the Nafion^®^ material, obtaining composites (10, 15, and 20 wt. % of modifier)—the proton conductivity of which reached twice the value relative to the base material. At the same time, the thermal resistance of the composite remained at the level of the indicator for Nafion, with a slight increase in water absorption. This approach is convenient in that it allows one to adjust the characteristics of membranes without affecting the mechanism of formation of the conducting channels.

In this regard, diamond frameworks in membranes with variations in diamond functionalization methods provide greater opportunities for targeted modification of the structural and conductive properties of proton exchange membranes through the organization of hybrid conductive channels involving the diamond surface and through the transport of protons along diamond chains at the diamond-polymer interface. In our work, it was possible to modify the mechanism of proton transport in order to improve the conductivity and mechanical properties, and increase the strength of the membrane material due to the special qualities of diamonds (chemically inert, durable, thermally stable, and resistant to ionizing radiation, as a result of its developed surface with a controlled composition and number of functional groups) compared with other nano-sized objects.

Due to their beneficial properties, nanodiamonds are also used in filtration membranes for nuclear technologies with requirements for radiation resistance in the materials [87]. Carboxylated nanodiamonds (CND) were used to modify polysulfone (Psf), which had limited radiation resistance (up to 100 kGy). Membranes with a CND fraction of 0.5% wt. withstood doses an order of magnitude higher (1000 kGy) [87], as the diamond particles adsorbed chemically aggressive products of water radiolysis [88]. The prospects for using diamonds to increase the thermal and radiation resistance and service life of filtration polymer membranes were discussed in review [89], in connection with the problems of modifying the surface of diamonds. Nanodiamond particles were silanized [90] to be incorporated into Psf membranes (up to 1 wt. %) to increase the hydrophilicity, water permeability, and porosity of the material with smaller void sizes. Through interfacial polymerization, a polyamide composite was obtained with diamonds that had reactive functional groups and a hydrophilic surface for binding to the matrix, increasing the wettability of the membrane surface and increasing the thermal stability [91].

When discussing the possibilities of using diamonds in membrane technologies, it should be noted that not only nanodiamonds, but also other carbon particles with a high specific surface area and acid groups, have been introduced into Nafion^®^ and Aquivion^®^ materials [92,93,94]. Multiwalled nanotubes (CNTs), deagglomerated detonation diamonds (DNDs), and nanocharcoal enhanced the conductivity of the Aquivion^®^ material [93]. CNTs (diameter 8–10 nm, specific surface area 276 m^2^/g) grown from the gas phase and treated with concentrated nitric acid were used. The synthesis of Aquivion^®^ composite membranes with oxidized carbon nanotubes was also performed [95]. The increase in proton conductivity was observed in such membranes, especially at a low humidity, which was also confirmed by experiments [93].

To modify the membranes, nanocharcoal (particle size 30–40 nm, specific surface area 1000 m^2^/g), obtained by igniting methane in a chlorine atmosphere, followed by purification in a nitrogen flow (1000 °C) and oxidation in air (300 °C), was used [93]. Sulfonated graphene oxide was incorporated into Nafion^®^ to improve the conductivity over a wide temperature range by reorganizing the conductive channels and increasing the proportion of bound water [94]. In samples of CNTs, nanocharcoal, and DND, the concentrations of acid sites on the particle surface were 0.75, 3.5, and 0.4 mmol/g [93]. Despite the low content of acid groups, diamonds provided the greatest effect for increasing conductivity at a low humidity [93]. At 12% humidity, the conductivity of the Aquivion material increased four times as a result of the addition of 0.4% DND. This was explained by the small size (5 nm) of particles which could enter membrane channels, changing their geometry and improving conductivity [93], according to the theory of [96].

These data, in connection with the results of our work, seem important, as they show that diamonds modify membrane structure due to their ability to self-organize in low-molecular and polymeric media. Suspensions of nanodiamonds in liquid polydimethylsiloxane were tested using broadband dielectric spectroscopy and X-ray scattering [97]. The conductivity and dielectric constant of the suspensions strongly depended on the chemical composition of the surface of the particles; polarization processes with temperature changes were of an activation nature, which indicated the structuring of diamonds [97]. The conclusion was confirmed by rheological and structural data for DND hydrosols and gels (particles 4–5 nm, 1–7 wt. %) with negative (ζ < 0) and positive (ζ > 0) electrokinetic potentials [98]. At DND fractions of 4–6 wt. % in experiments with rotational viscometry, X-ray scattering, NMR, and cryo-electron tomography, a sol-gel transition with viscosity hysteresis and a thixotropic effect was discovered, which was explained by the formation of a network during the interaction of diamonds [98]. The aggregation of diamonds was enhanced as a result of the grafting of lanthanide atoms (Eu and Gd) to the surface of particles, which led to the formation of branched fractal structures with a dimension of 2.4 and a scale of 40–1500 nm, according to neutron scattering data in aqueous dispersions of modified diamonds [99].

As our results have shown, fractal patterns of ordering for ensembles of diamond particles are especially important for the formation of composite membranes with a diamond modifier. In this case, it is necessary to regulate the state of the surface of diamond particles, as well as the number and type of functional groups (proton donors) in order to set the particle potentials that determine their interaction and ordering in membranes. The results of quantum chemical modeling of charges on hydrogen atoms of groups (H, OH, and COOH) on the surface of diamonds showed that a hydrogen atom in the OH group had the highest charge [1,100,101,102,103,104]. The same methods were used to calculate the electronic structure of SO_3_H groups in Nafion^®^ and Aquivion^®^ monomers in order to evaluate the influence of the charge states of atoms in ionic groups on the proton mobility [101].

The developed modeling approaches [100,101] allowed for optimizing the strategy for creating composite membranes with nanodiamonds and other forms of nanocarbon in connection with various applications (chemical energy sources—fuel cells, redox batteries, reverse electrodialysis devices, and lithium-ion batteries) [1]. Although the possibilities of perfluorinated membrane technologies have not been exhausted, composites based on other polymers, in particular polybenzimidazole, are being created. Such composites showed the highest conductivity with a significant proportion of silicon dioxide in propylimidazoline groups (10 wt. %), as at low concentrations of the modifier, the permeability of the membranes to hydrogen decreased [102].

Along with this, interest remains in the development of membranes made from perfluorinated sulfocationic polymers (thermally initiated polymerization at high pressure) [103]. The resulting samples, tested at temperatures of 21 and 79 °C, had conductivities of 57 and 114 mS/cm, respectively, which is higher than the commercial Nafion^®^ material, reespectively. Research is being developed [104] in the field of perfluorinated sulfonated cation-exchange membranes, differing in the length of the side chain and the proportion of fragments with ether groups. The material MF-4SK (Plastpolymer, St. Petersburg, Russia) in protic and potassium formed when modified with membrane foil (Mega, Czech Republic) and phosphate-modified zirconium dioxide [105] demonstrated a strong increase in conductivity and selectivity. Recently created [106] polymer membranes (Nepem-117) in Li+ form, when saturated with polar aprotic solvents (dimethylformamide, dimethyl sulfoxide, dimethylacetamide, and mixtures of solvents), showed an increase in ion mobility as the degree of membrane solvation increased (NMR data), which exceeded the characteristics of Nafion^®^ membranes with aprotic solvents [106].

At the same time, the variety of possibilities for modifying membranes with various nanoparticles is much wider, especially taking into account the options for grafting functional groups onto their surface. Thus, due to phosphonic groups grafted to the polymer in hybrid membranes of N-phosphorylated polybenzimidazole with silica (2–20 wt. %, particles with sizes of 3–5 and 20–60 nm), as a result of additional hydration, the increase in the proton conductivity of the composites occurred at a sufficiently high temperature (130 °C) [107]. Considerable attention has been paid to studying the influence of the acid-base properties of inorganic particles on the physicochemical and transport properties of ion-exchange membranes [108]. Particles of Zr, Ti, and Si oxides were synthesized in the pores and channels of the membranes, which, depending on the acid-base properties, increased or decreased water absorption, conductivity, and selectivity. Cross-linking of ion-exchange membranes using ZrO_2_ particles made it possible to increase the swelling, conductivity, and selectivity of salt-permeable membranes in the Na^+^ form [108]. The introduction of sulfonated zirconium oxide into perfluorinated cation-exchange membranes MF-4SK made it possible to increase their conductivity at room temperature by four times, while reducing gas permeability to hydrogen by a factor of three [109]. Hybrid ion-exchange membranes were obtained by directly synthesizing amorphous zirconium phosphate (0.5–24 wt. %) in the pores of the RALEX^®^ CM matrix, which led to the displacement of water from the pores and an increase in the number of cation transfers, increasing selectivity for monovalent ions [110].

Thus, oxides of metals and other elements are mainly used in hybrid membranes, where particles are introduced into matrices or synthesized in their pores while carbon nanoparticle applications are just beginning to develop. The results of our work show the abilities and prospects for introducing diamonds into membrane technologies using various methods of modifying the surface of diamonds, regulating the type and number of grafted functional groups, surface potentials of particles, and, ultimately, forms of structuring diamonds in polymer matrices to achieve increased conductivity, moisture absorption, and mechanical and temperature stability for hybrid membranes. The original results obtained can be compared with a detailed analysis of studies in the field of membranes based on perfluorinated copolymers, given in the review of [5]. The main trends in the development of perfluorinated membrane technologies are presented in a series of original studies and reviews devoted to the improvement of functional properties of these materials through modification with various nanoparticles [111,112,113,114,115,116,117,118].

## 4. Conclusions

As a result of the electrophysical and structural studies of composites of perfluorinated Aquivion^®^-type copolymers modified with detonation nanodiamonds in the process of membrane film preparation from liquid mixtures of components, the mechanisms of action of diamond particles on the network of ion channels of the copolymer were revealed, and the patterns of formation of the conducting diamond-polymer interface were revealed. It was established that DND Z+ particles with a hydrogen-saturated surface were most effective at increasing the conductivity of membranes, providing them with a positive potential during hydration and ensuring the interaction of attraction to the negatively charged sulfonic acid groups of the copolymer, facilitating the integration of diamonds into the polymer matrix. 

Hydrophilic DND Z+ was compatible with the copolymer and did not interfere with its structuring with the formation of ion channels. Moreover, DND Z+ particles created additional hybrid conductivity channels with ionic groups of the copolymer, which allowed for a stable increase in membrane conductivity (~20–30%) under heating conditions from 20 to 50 °C with varying diamond contents in the membranes (0.5–5.0 wt. %). The positive effect was due to a framework of branched chain diamond structures with a fractal dimension of ~2.2–2.4 created in the copolymer matrix. The hydrophilic surface of diamonds, saturated with hydrogen, attracted ionic groups of the polymer, forming a conducting interface, where the diamond facets served as binding centers for the adjacent ion channels of the copolymer, which contributed to better water saturation of the membranes and ensured the stability of the channel network.

Compared with DND Z+, DND Z− particles with grafted carboxyls, which imparted a negative charge to the diamond surface upon hydration, were less likely to create additional conduction channels by integrating similarly charged ionic groups of the copolymer into them. At the same time, in composites with DND Z−, the diamond structures had a reduced fractal dimension of ~1.5–1.7, which indicated linear chains with an excluded volume, and such chains contributed less to the construction of a diamond framework in the matrix. For these reasons, the conductive properties of these composites were inferior to those of the membranes with DND Z+.

As an alternative, Aquivion^®^-type composites with fluorinated diamonds were prepared, which interacted primarily with the fluorocarbon chains of the copolymer. In this case, the self-organization of copolymer chains with the formation of ion channels was disrupted. Backbone hydrophobic chains of the copolymer were adsorbed on the diamond facets, and the adjacent ionic groups at the ends of the side chains could only form isolated multiplets due to steric restrictions. As a result, a small addition of DND-F (1 wt. %) caused a 4-fold decrease in membrane conductivity.

A comparative analysis of the electrical and structural properties of Aquivion^®^-type composites modified with the detonation nanodiamonds with various functional groups on the surface demonstrated the advantages of using DND Z+ detonation nanodiamonds to strengthen and stabilize the network of ion channels of proton-conducting membranes, which provides a significant effect for increasing the conductivity of the composite at elevated temperatures and shows the possibilities for the application of these new materials in fuel cells.

## Data Availability

Data is contained within the article and Supplementary Materials.

## Supplementary Materials

The following supporting information can be downloaded at: https://www.mdpi.com/article/10.3390/membranes13110850/s1, Figure S1: TEM pattern for dried DND Z+ hydrosol; Figure S2: Microscopy patterns for the pristine membrane without diamonds (AFM, a), (SEM, b) and compositional membranes with 2 wt. % of DND Z- (AFM, c), 5 wt. % of DND Z+ (SEM, d), 1.9 wt. % of DND-F (SEM, e); Figure S3: Stress-strain diagrams for samples of the Aquivion^®^-type membranes with DND-F; Table S1: Composites of the Aquivion^®^-type membranes with DND-F. References [119,120] are cited in the supplementary materials.
