# Supplementary material for: Electrochemical Properties and Structure of Membranes from Perfluorinated Copolymers Modified with Nanodiamonds
Source: Membranes (Basel). 2023 Oct 25;13(11):850. doi: 10.3390/membranes13110850 (PMC10673602; doi:10.3390/membranes13110850)
Supplement: Supplementary file 1 [file membranes-13-00850-s001.zip › membranes-2641562-supplementary.pdf]

Supplementary Information

I. Characterization of diamonds.

Typical TEM picture of diamond aggregates formed by hydrosol drying is shown in Figure S1 (analytical electron microscope Zeiss Libra 200FE).

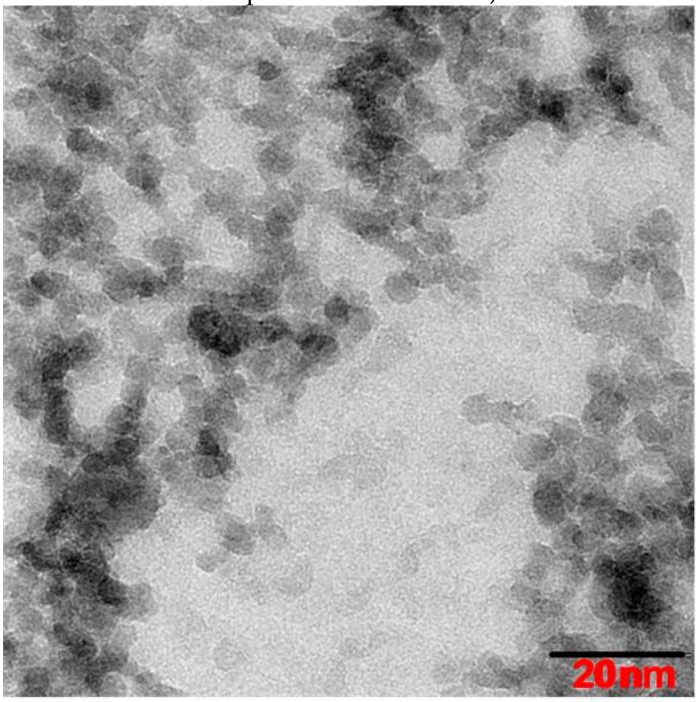

Figure S1. TEM pattern for dried DND Z+ hydrosol.

II. AFM and SEM characterization of membranes.

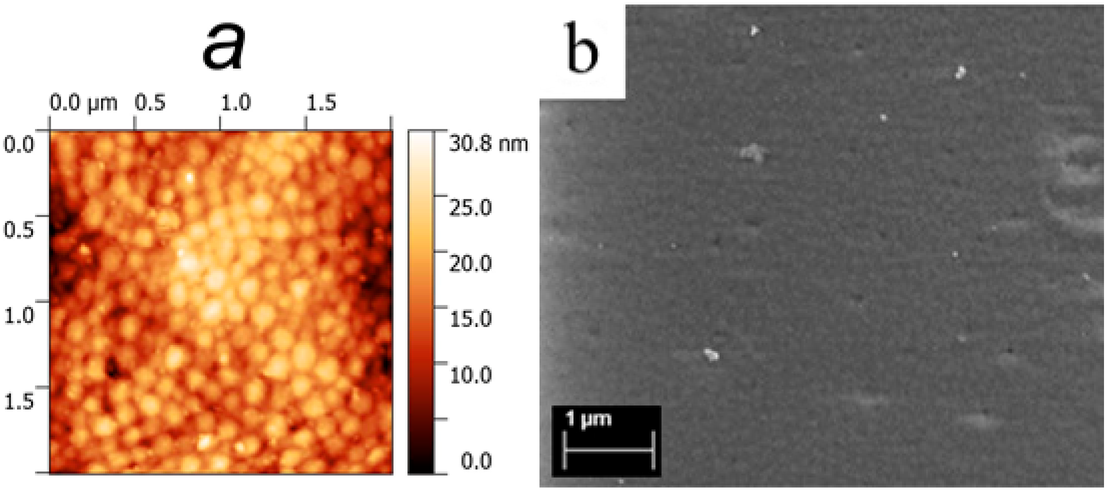

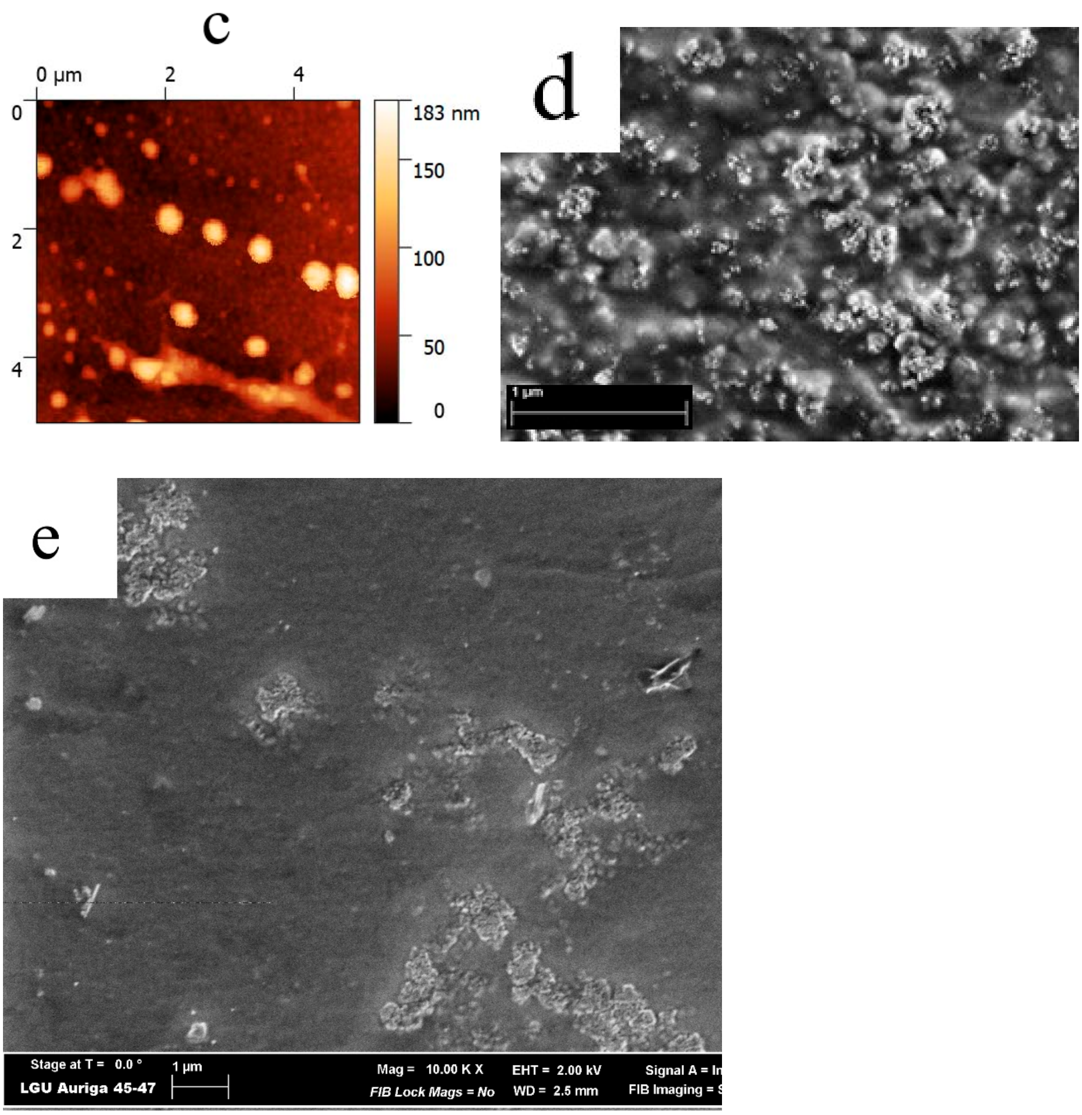

Figure S2. Microscopy patterns for the pristine membrane without diamonds (AFM, a), (SEM, b) and compositional membranes with 2 wt. % of DND Z- (AFM, c), 5 wt. % of DND Z+ (SEM, d), 1.9 wt. % of DND-F (SEM, e).

III. Mechanical tests of Aquivion®-type membranes with DND-F.

Mechanical tests were carried out for Aquivion®-type membranes in acidic form under conditions of uniaxial tension at a constant speed at a constant temperature (20°C) and a given humidity RH ~ 50-60%. Young's modulus (E), the stress of transition through the plastic limit (σ_p), deformation and stress until destruction of the material (ε_D, σ_D) were determined.

The Aquivion + DND-F composites with fluorinated diamonds (Table 1, Fig.3) had elastic moduli of 230-310 MPa, strain to destruction of 110-150% and showed a transition through the plastic limit at the inflection point of the deformation curve when stretched by ~ 5-10% with subsequent strain hardening of the material.

Table S1. Composites of the Aquivion®-type membranes with DND-F.

№	Content, thickness (µm)	RH, %	E, MPa	σ_p, MPa	σ_D, MPa	ε_D, %

0	Polymer without DND, (70-74)	49	228 ± 11	14 ± 1	26 ± 2	321 ± 23
1	Polymer + 0.25 % DND-F, (54-56)	49	237 ± 3	12.1 ± 0.5	14.3 ± 0.7	112 ± 15
2	Polymer + 0.5 % DND-F, (44-49)	51	246 ± 10	12.8 ± 0.4	15.1 ± 0.5	119 ± 6
3	Polymer + 1.0 % DND-F, (55-60)	51	229 ± 8	15.0 ± 0.2	19.4 ± 0.4	149 ± 3
4	Polymer + 1.9 % DND-F, (52-57)	50	312 ± 14	15.5 ± 0.6	18.7 ± 0.9	123 ± 6

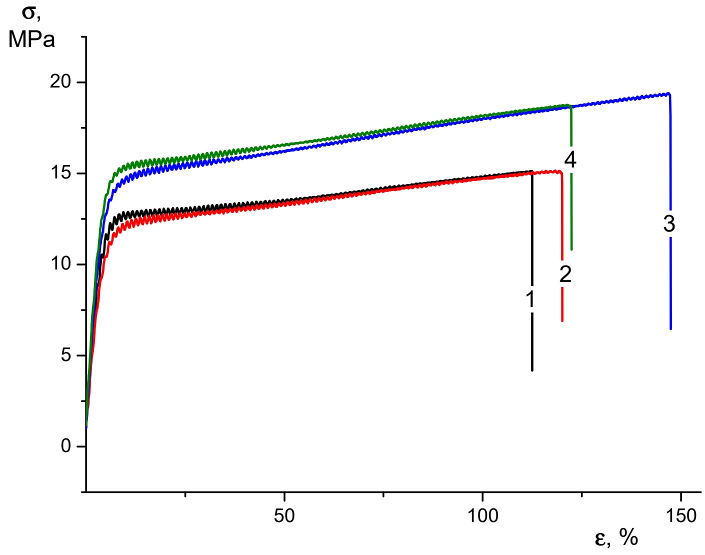

Figure S3. Stress-strain diagrams for samples of the Aquivion®-type membranes with DND-F.

Modification of the copolymer with DND-F diamonds caused an increase in the elastic modulus of the material, but with a small content of diamonds (0.25 wt. %) the plastic limit decreased and then recovered when the matrix was enriched with diamonds. A similar effect of a sharp decrease in fracture stress σ_D was observed with the introduction of 0.25 wt. % diamonds and its subsequent growth as a result of the addition of a modifier. Fracture strains remained low (110-150 %) at diamond concentrations of 0.25-1.9 wt. % (Table S1). As it turned out, fluorinated diamonds served to some extent as a plasticizer in the polymer matrix, which was also observed for sulfonated diamonds in contrast to composites with DND Z+, DND Z-.

In our recent work [11] such tests were performed also for pure Aquivion type membrane and the composites with sulfonated diamonds (0.25-5.0 wt. %). The results have shown for pristine membrane the elastic modulus E ~ 230 MPa and for the composites quite similar moduli E ~ 210-240 MPa. Membranes' modification with diamonds did not cause dramatic changes in the characteristics of material: σ_Y ~ 10-14 MPa, σ_T ~ 15-25 MPa [11]. Our results for pristine Aquivion type membrane are in good agreement with the characteristics of Aquivion films with similar equivalent weight [119]. Authors [119] used SSC-PFSA membrane (Aquivion) provided by Solvay (EW= 850, dry membrane thickness of 100 μm). This dry material (25°C) had the elastic modulus E ~ 600 MPa, while after water absorption it comes down to E ~ 150 MPa.

Mechanical tests of Aquivion + DND Z- were discussed in our works [17,31,120], data for Aquivion + DND Z+ were published in [10, 120].

References

10. Primachenko, O.N.; Kulvelis, Y.V.; Odinokov, A.S.; Glebova, N.V.; Krasnova, A.O.; Antokolskiy, L.A.; Nechitailov, A.A.; Shvidchenko, A.V.; Gofman, I.V.; Marinenko, E.A.; Yevlampieva, N.P.; et al. New Generation of Compositional Aquivion®-Type Membranes with Nanodiamonds for Hydrogen Fuel Cells: Design and Performance. *Membranes* **2022**, *12*, 827. https://doi.org/10.3390/membranes12090827.

11. Shvidchenko, A.V.; Odinokov, A.S.; Primachenko, O.N.; Gofman, I.V.; Yevlampieva, N.P.; Marinenko, E.A.; Lebedev, V.T.; Kuklin, A.I.; Kulvelis, Y.V. Improving PFSA Membranes Using Sulfonated Nanodiamonds. *Membranes* **2023**, *13*, 712. https://doi.org/10.3390/membranes13080712.

17. Kulvelis, Y.V.; Primachenko, O.N.; Odinokov, A.S.; Shvidchenko, A.V.; Bayramukov, V.Y.; Gofman, I.V.; Lebedev, V.T.; Ivanchev, S.S.; Vul, A.Y.; Kuklin, A.I.; et al. Composite proton-conducting membranes with nanodiamonds. *Fuller. Nanotub. Carbon Nanostructures* **2020**, *28*, 140–146. https://doi.org/10.1080/1536383X.2019.1680981.

31. Primachenko, O.N.; Kulvelis, Y.V.; Lebedev, V.T.; Odinokov, A.S.; Bayramukov, V.Y.; Marinenko, E.A.; Gofman, I.V.; Shvidchenko, A.V.; Vul, A.Y.; Ivanchev, S.S. Perfluorinated Proton-Conducting Membrane Composites with Functionalized Nanodiamonds. *Membr. Membr. Technol.* **2020**, *2*, 1–9. https://doi.org/10.1134/S2517751620010060.

119. Zhao, Q.; Benziger, J. Mechanical properties of perfluoro sulfonated acids: The role of temperature and solute activity. *J. Polym. Sci. B, Polymer Physics* **2013**, *51*, 915–925. https://doi.org/10.1002/polb.23284.

120. Kulvelis, Y.V.; Primachenko, O.N.; Gofman, I.V.; Odinokov, A.S.; Shvidchenko A.V.; Yudina, E.B., Marinenko, E.A.; Lebedev, V.T.; Vul, A.Y. Modification of the mechanism of proton conductivity of the perfluorinated membrane copolymer by nanodiamonds. *Russ. Chem. Bull. Int. Ed.* **2021**, *70*, 1713–1717. https://doi.org/10.1007/s11172-021-3274-4.

Acknowledgments: The authors thank IBR-2 reactor staff (JINR, Dubna) for providing SANS experiments, Analytical Resource Centre of St Petersburg University and N.P. Yevlampieva for the help in TEM and SEM data obtaining, V.Yu. Bairamukov (PNPI) for the help in AFM experiments, I.V. Gofman (IMC RAS) for performing the mechanical tests and I.N. Ivanova (PNPI) for technical help.
